# CHH methylation is recruited to gene-proximal transposable elements during repeated drought stress

**DOI:** 10.64898/2026.07.14.738593

**Published:** 2026-07-20

**Authors:** Lily D. Peck, Victoria L. Sork

## Abstract

Trees experience decades-to-centuries of environmental change within a single lifetime, requiring molecular mechanisms that enable rapid physiological and transcriptional adjustment without genetic adaptation across generations. Increasing drought frequency provides one important example of the environmental challenges faced by long-lived species. DNA methylation is a candidate regulator of such responses, but whether environmentally induced methylation primarily protects the genome, regulates nearby transcription, or both, remains an outstanding question. To address this question, we integrated methylome and transcriptome data from valley oak ( *Quercus lobata* ) seedlings exposed to drought and well-watered conditions. Drought conditions induced widespread and dynamic CHH methylation that repeatedly targeted the same gene-proximal transposable elements (TEs) across successive drought exposures despite turnover of individual methylated cytosines. This response was concentrated within specific TE families, indicating targeted recruitment of CHH methylation across the genome. Genes associated with CHH-methylated upstream TEs showed increased transcription under drought and were enriched for drought-response pathways, including abscisic acid signaling, cuticle and wax biosynthesis and cell wall remodelling. Nonetheless, the magnitude of transcriptional activation declined with increasing CHH methylation, indicating a graded regulatory effect rather than binary silencing. Despite little overall change in the TE transcriptome, greater CHH methylation was specifically associated with reduced expression of intragenic TEs, consistent with maintenance of local TE repression. These findings support a model in which repeated drought consistently recruits CHH methylation to reproducible gene-proximal TEs, where it is associated with maintenance of local TE repression despite continued activation of neighboring stress-responsive genes. Increasing CHH methylation is associated with progressively weaker transcriptional responses, suggesting that high levels of CHH methylation may simultaneously suppress TE activity and constrain nearby gene expression. Such a mechanism may influence how long-lived trees repeatedly adjust transcriptional responses to fluctuating climates throughout their lifespan.

**Teaser:**

Genome protection during drought may carry an associated cost to stress-responsive gene expression.

## Full Text Availability

The license terms selected by the author(s) for this preprint version do not permit archiving in PMC. The full text is available from the preprint server.

