# Repeated drought induces a reproducible DNA methylation response associated with gene expression in *Quercus lobata*

**DOI:** 10.64898/2026.07.14.738593

**Published:** 2026-09-07

**Authors:** Lily D. Peck, Victoria L. Sork

**Affiliations:** 1 Department of Ecology and Evolutionary Biology, University of California Los Angeles; 2 Institute of the Environment and Sustainability, University of California Los Angeles

**Keywords:** Biological sciences and evolution, Stress, climate, epigenetics, genome regulation, transposable elements

## Abstract

Long-lived trees must continually adjust to environmental change and face sustained climatic shifts over their lifetimes. One increasingly important challenge is the rising frequency of drought caused by climate change. Environmentally responsive DNA methylation is widespread in plants, but whether it contributes to gene expression during environmental stress remains unclear, particularly in long-lived trees. Here, we integrated long read methylomes and transcriptomes from valley oak (*Quercus lobata*) seedlings exposed to repeated drought and well-watered treatments. Repeated drought induced a reproducible DNA methylation response that repeatedly targeted the same genomic regions despite turnover of individual methylated sites. These repeatedly targeted regions were transposable elements (TEs) located near genes. Genes adjacent to CHH-methylated TEs were enriched for core drought-response pathways, including abscisic acid signaling, osmotic adjustment and cell-wall remodeling, and remained transcriptionally activated under drought. However, higher CHH methylation levels were associated with progressively smaller transcriptional responses, suggesting that environmentally responsive DNA methylation influences how strongly drought-response genes are activated rather than simply switching them on or off. At the same time, greater CHH methylation was associated with continued repression of nearby TEs, suggesting that this response may simultaneously regulate gene activity while maintaining genome stability. Together, these findings identify a reproducible genome-regulatory response associated with repeated environmental stress in a long-lived tree. By repeatedly targeting the same genomic regions despite turnover of individual sites, this response provides a framework for how long-lived trees repeatedly adjust gene expression while maintaining genome stability during environmental change.

## Introduction

Long-lived trees experience decades to centuries of environmental change while remaining rooted in the same place throughout their lives. Unlike annual plants or short-lived perennials, which can adapt across generations through natural selection, individual trees must repeatedly adjust their physiology and gene expression to changing conditions throughout their lifespan (1, 2). How these repeated molecular responses are coordinated remains poorly understood. This question is becoming increasingly important because many trees living today established under climate conditions that differ from those they will experience in the future (2, 3). Drought is a major source of stress for plants because water limitation disrupts core physiological processes including photosynthesis, growth, and carbon allocation (4–7). Under climate change, droughts are predicted to become increasingly frequent and severe (8), and understanding the molecular mechanisms that enable repeated environmental responses has become central to predicting forest resilience under climate change (9).

In plants, physiological responses to environmental stress are coordinated through changes in gene expression (10). These transcriptional responses are determined not only by promoter and cis-regulatory sequences but also by chromatin state and the broader genomic context surrounding genes (11–14). Gene-proximal transposable elements (TEs) are increasingly recognized as important components of this regulatory landscape because they can influence chromatin accessibility and nearby transcription while remaining tightly controlled to preserve genome stability (15). Understanding how environmental stress reshapes this regulatory landscape is therefore central to understanding how plants repeatedly adjust gene expression (16).

DNA methylation is a major component of plant chromatin regulation, influencing transposable element silencing, chromatin accessibility and gene expression (17, 18). Although methylation occurs in CG, CHG, and CHH sequence contexts (where H=A, T, or C), CHH methylation is particularly dynamic because it depends upon continual targeting through the RNA-directed methylation pathway (19–23). Consequently, CHH methylation has emerged as one of the most environmentally responsive components of the plant methylome and is enriched at transposable elements where it may influence nearby transcription while maintaining TE repression (17, 24). Rather than acting as a simple on/ off switch, accumulating evidence suggests that DNA methylation affects gene expression in quantitative ways, modulating expression levels (18, 25–27), raising the possibility that environmentally responsive CHH methylation contributes to fine-scale adjustment of stress-responsive gene expression.

Environmentally associated DNA methylation is widely documented in plants (19, 28–31), yet it remains unclear how epigenomic responses contribute to transcriptional regulation during repeated environmental stress. Studies in forest trees have documented drought-associated methylation changes (32–35), and other plant studies have implicated transposable elements in shaping stress-responsive transcription (36). However, these processes have rarely been examined together, leaving unresolved whether environmentally responsive methylation is associated with gradients of nearby gene expression or simply reflects a downstream consequence of stress. Recent comparative analyses in *Quercus lobata* suggest that CHH methylation, TE distribution, and gene expression are linked (37), but the dynamics of this relationship during repeated drought need to be determined.

To understand how long-lived organisms repeatedly respond to fluctuating environments, we investigated one type of regulatory mechanism at the molecular level that may coordinate these responses. Specifically, we therefore asked whether repeated drought induces a reproducible DNA methylation response that is associated with gradients of gene expression in valley oak (*Q. lobata* Née). Using matched long-read methylomes and transcriptomes collected across repeated drought and well-watered treatments, we tested whether repeated drought (i) induces reproducible CHH methylation, (ii) concentrates this response at gene-proximal transposable elements, and (iii) associates this regional methylation with gradients of nearby drought-responsive gene expression. Our results identify a reproducible DNA methylation response linked to transcriptional responses during repeated drought, informing how long-lived trees coordinate rapid environmental responses while maintaining genome stability.

## Results

### Oak seedlings show reduced transpiration under drought stress

**Figure 1.**
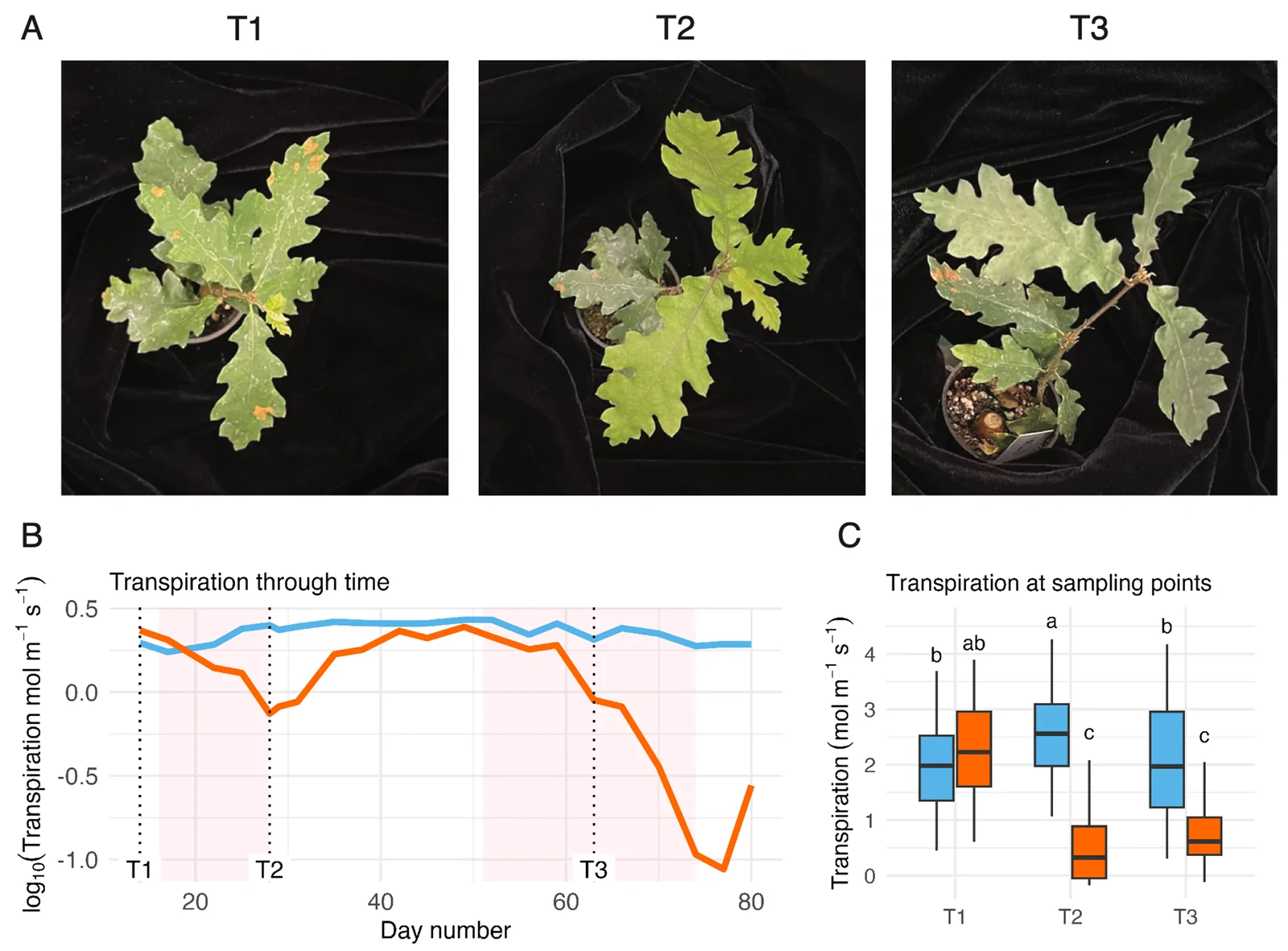
Transpiration in *Quercus lobata* seedlings under drought treatment. **(A)** Representative seedling before drought (T1), after 10 days of drought (T2), and following recovery and a second 10-day drought (T3). We observed a breakdown in chlorophyll from T2 to T3 as drought progressed. (**B)** Time course of transpiration for 80 seedlings across well-watered (blue) and drought (orange) treatments. Values represent means across seven maternal families, with measurements taken at midday. Shaded regions indicate drought periods. Vertical dashed lines mark sampling time points (T1-T3). (**C)** Transpiration rates at T1, T2, and T3 for 80 seedlings across well-watered (blue) and drought (orange) treatments. Boxes show interquartile ranges, center lines indicate medians calculated across seven families. Different letters indicate significant differences among treatment x timepoint groups based on Tukey-adjusted pairwise comparisons (*P* < 0.05).

Our observations of 80 *Quercus lobata* seedlings from seven maternal trees (hereafter referred to as families, Figure S1A) growing in drought and well-watered treatments, showed increasing differences in physiological measures across three time points: pre-drought (T1); after 10 days of drought (T2); and following recovery and a second 10-day drought (T3; Figure 1A and Figure S2). Transpiration declined rapidly in response to water limitation, indicating that this measure captured the physiological impact of drought stress. Following the onset of drought, transpiration in drought-treated seedlings decreased relative to well-watered controls and remained suppressed throughout the experiment (Figure 1B). This divergence was most pronounced during the drought periods and did not fully recover following rewatering. At the discrete sampling points, transpiration was significantly lower in drought-treated seedlings than controls at both T2 and T3 (Tukey-adjusted pairwise comparisons, *P* < 0.001 for both), whereas no difference was detected prior to drought at T1 (*P* = 0.462; Figure 1C).

Within drought-treated seedlings, transpiration declined significantly from T1 to both T2 and T3 (*P* < 0.001 for both) but did not differ between T2 and T3 (*P* = 0.902), providing no evidence of a differential physiological response between the first and second drought periods. Across the experiment, transpiration was significantly influenced by treatment (linear mixed-effects model: *F*_1,69_ = 26.6, *P* = 2.26 × 10^−6^), time (*F*_2,150_ = 23.5, *P* = 1.34 × 10^−9^), and their interaction (*F*_2,150_ = 54.7, *P* < 0.001), whereas family had no detectable effect (*F*_6,69_ = 1.33, *P* = 0.254), indicating a consistent physiological response to drought across the seven maternal families used in this experiment. These consistent reductions in transpiration confirm that drought treatment imposed sustained physiological stress, providing a framework to interpret genome-wide changes in DNA methylation.

### Drought induces context-specific changes in DNA methylation

Using eight seedlings from two families, we generated 24 Nanopore PromethION methylomes across three time points (T1-T3), comprising four genetically distinct seedlings per family under drought and well-watered conditions (Figure S1B). Methylomes were similar among seedlings at T1 (Figure S3). Genome-wide methylation landscapes remained broadly stable across treatments. CG and CHG sites were predominantly highly methylated, whereas CHH methylation was sparse, typically below 10% (Figures S4, S5A). Patterns of methylation across gene bodies, introns, and flanking regions (Figures S5B–D) closely matched those previously observed in *Q. lobata* (37, 38) and other angiosperms (39).

**Figure 2.**
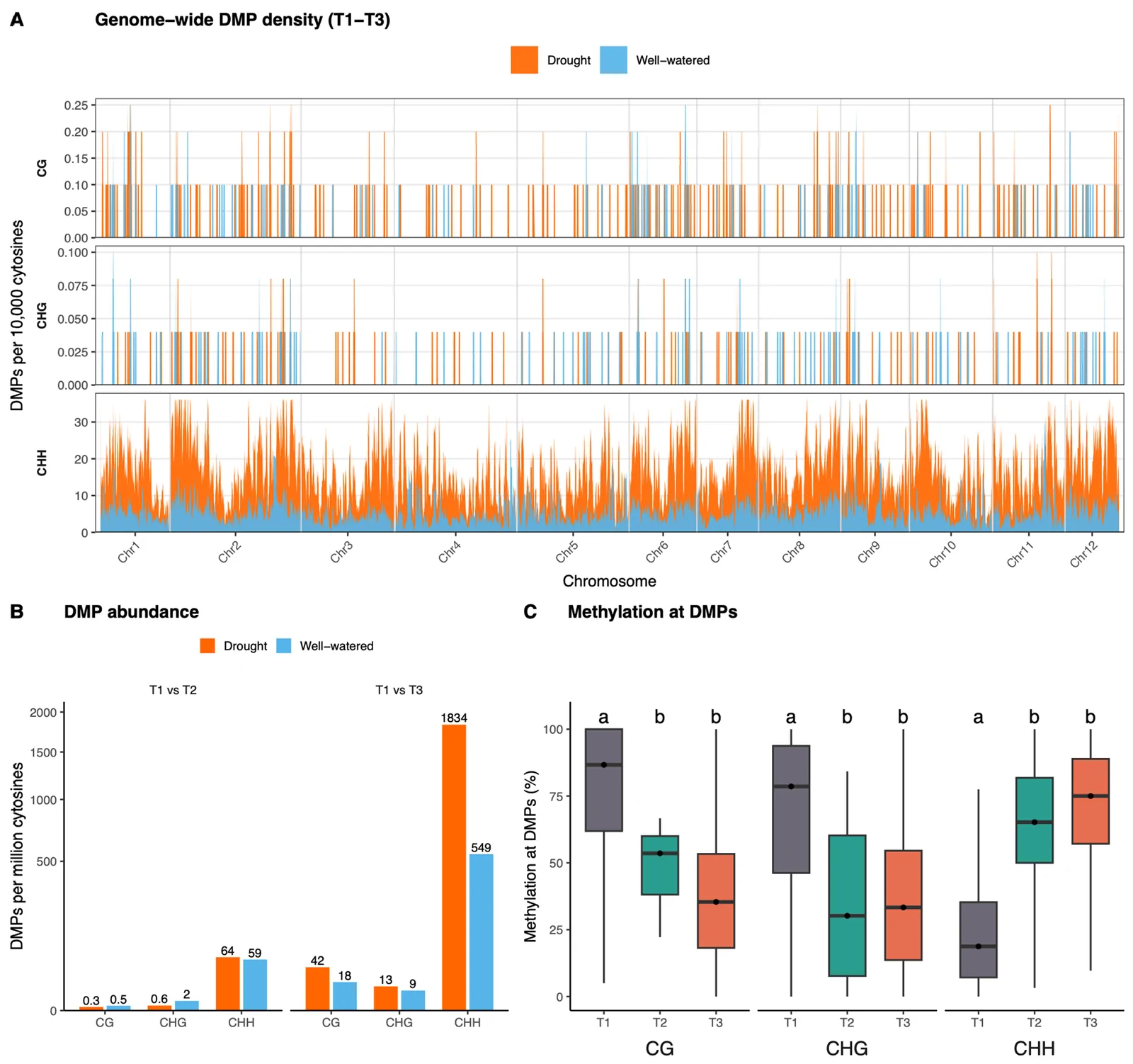
Distribution, abundance, and dynamics of drought-associated differentially methylated positions (DMPs). DMPs shown represent concordant methylation changes identified in all four drought-treated seedlings (or all four well-watered controls for the control catalogue) following coverage filtering and direction matching. **(A)** Genome-wide density of T1–T3 DMPs across the twelve chromosomes for each sequence context (CG, CHG, CHH), expressed as DMPs per 10,000 genomic cytosines in 100-kb windows and smoothed using a 1-Mb rolling mean. Well-watered controls (blue) are overlaid on drought-treated seedlings (orange). **(B)** DMP abundance for the T1–T2 and T1–T3 contrasts, expressed as DMPs per million genomic cytosines to account for differences in the genomic representation of each sequence context. **(C)** Methylation levels at drought-associated DMPs across time points (T1–T3) in the four drought-treated seedlings for each sequence context. Boxes indicate interquartile ranges, horizontal lines indicate medians, and points indicate mean methylation values. Different letters denote significant differences among time points within a sequence context (Benjamini–Hochberg-adjusted pairwise comparisons of per-individual mean methylation).

Despite overall stability in genome-wide methylation landscapes, drought induced extensive differential methylation at individual cytosines. Concordant differentially methylated positions (DMPs), defined as loci showing the same methylation response in all four treatment seedlings and with the drought-associated DMPs absent from their well-watered controls, were distributed across all twelve chromosomes and were consistently more abundant in drought-treated seedlings than in well-watered controls, particularly in the CHH context (Figure 2A). Because these DMPs were concordant across biological replicates, they represent reproducible drought-associated methylation changes rather than individual-specific variation. When normalized for genomic cytosine abundance, CHH DMP levels were similar between treatments at T1-T2 (64 versus 59 DMPs per million cytosines) but diverged by T1-T3, with substantially more CHH DMPs in drought-treated seedlings than controls (1,834 versus 549 DMPs per million cytosines, Figure 2B). In contrast, CG and CHG DMPs remained rare in both treatments, although modest increases were observed under drought at T1-T3 (CG: 42 versus 18; CHG: 13 versus 9 DMPs per million cytosines). Additionally, CHH sites were strongly enriched for differential methylation, whereas CG and CHG sites were depleted relative to genomic expectations (Figure S6).

The drought-induced CHH methylation response was highly dynamic at individual cytosines but repeatedly targeted the same genomic regions across successive drought exposures. Overlap between CHH DMPs at single-cytosine resolution was limited, with ~25% of first exposure CHH DMPs recurring within ±10 bp at T1-T3. However, overlap increased rapidly as the positional matching window widened, indicating that repeated drought frequently methylated nearby rather than identical cytosines (Figure S7). At the broader scale of individual TEs, approximately half of the TEs carrying CHH DMPs at T1-T2 also carried them at T1-T3, representing a 5-6x fold enrichment relative to a genomic CHH background permutation null (*P* < 0.001). This enrichment was consistent across all four drought individuals. No overlap was observed for CG or CHG DMPs. These results indicate that repeated drought conditions consistently target the same TEs, while the precise cytosines acquiring CHH methylation vary within those elements. Consistent with this dynamic response, methylation levels at CHH DMPs diverged progressively through time and were almost exclusively hypermethylation events, whereas CG and CHG DMPs were predominantly hypomethylation events (Figure 2C, Table S1).

These results demonstrate that drought induces a highly context-specific CHH methylation response that is both reproducible across individuals and dynamic through time. This response accumulates over time, emerges primarily following repeated drought exposure, and is driven largely by newly induced methylation events rather than persistent early changes. Similar stress-associated CHH hypermethylation has been reported in rice and Arabidopsis (20, 21), as well as accompanied with a dual loss of CG and CHG maintenance methylation in the resurrection plant, *Boea hygrometrica* (40). In short, these data suggest that environmentally responsive CHH methylation may represent a shared component of plant drought responses across species. To determine why repeated drought consistently targeted the same TEs, we next examined the genomic distribution of drought-associated DMPs.

### Drought-associated CHH methylation preferentially targets transposable elements near genes

**Figure 3.**
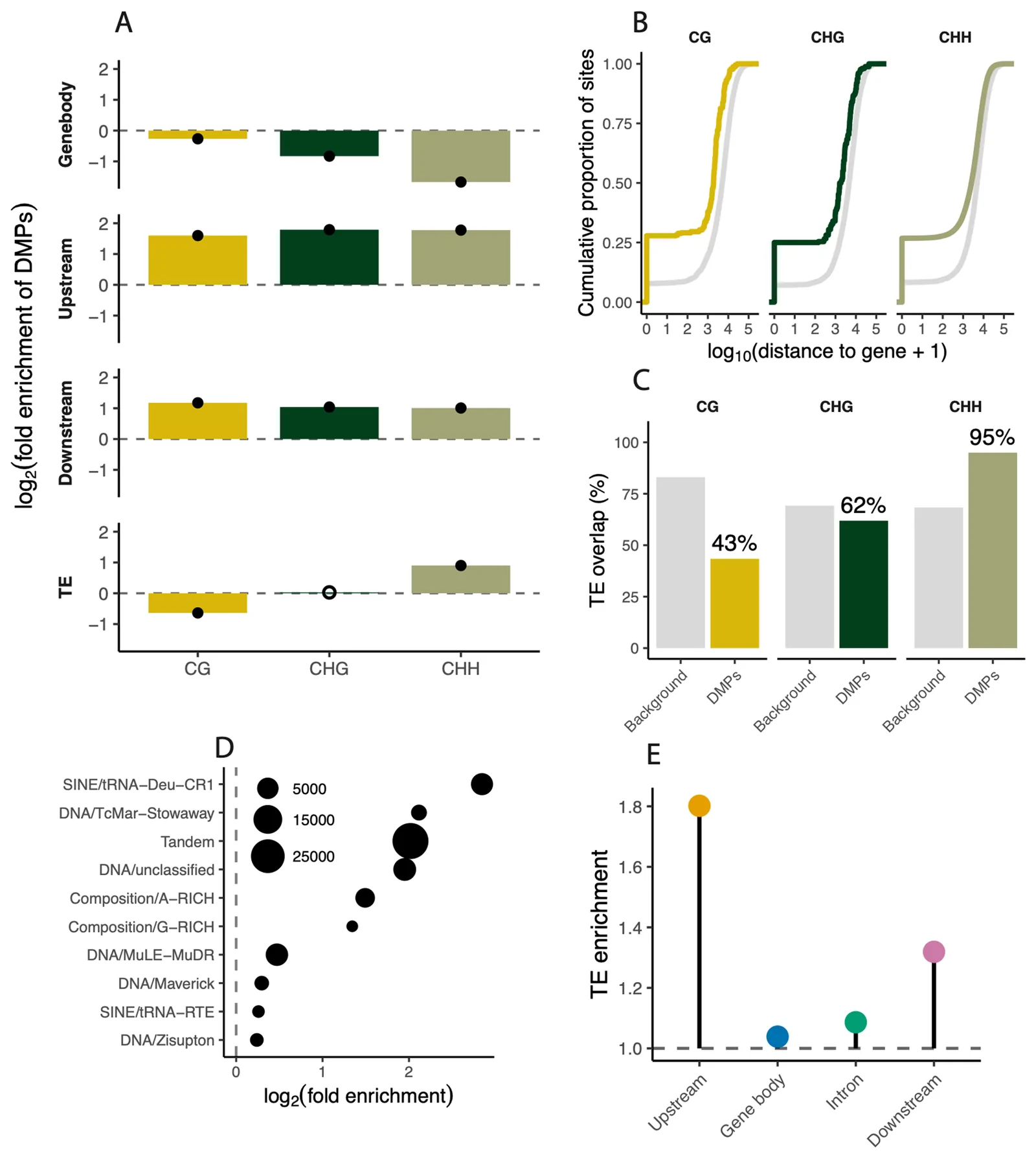
Genomic distribution and transposable-element-association of drought-responsive differentially methylated positions (DMPs). **(A)** Enrichment of DMPs across genomic features, shown as log2(fold enrichment) relative to the genomic distribution of cytosine sites in each sequence context. Positive values indicate enrichment and negative values indicate depletion. Filled points denote significant enrichment or depletion (Benjamini-Hochberg adjusted *P* < 0.05); open points indicate non-significant enrichment. **(B)** Cumulative distribution of distances from DMPs (colored) and all genomic cytosine sites (grey) to the nearest upstream gene flanking region. Distances are shown as log_10_(distance +1). **(C)** Proportion of DMPs and genomic background cytosine sites overlapping annotated transposable elements (TEs) across CG, CHG and CHH sequence contexts. Bars indicate the percentage of sites overlapping TE annotations. **(D)** Significant enrichment of CHH DMPs across TE superfamilies, ranked according to degree of enrichment and shown as log2(fold enrichment) relative to genomic TE abundance. Only superfamilies with significant enrichment (FDR < 0.05) are shown. Point size indicates the number of CHH DMPs assigned to each superfamily. **(E)** Distribution of TE copies from CHH DMP-enriched families across gene features. Upstream and downstream regions are within 1.5 kb from the gene. Points show odds ratios from Fisher’s exact tests relative to genomic expectations, with error bars indicating 95% confidence intervals.

To determine where drought-induced methylation changes occurred within the genome, we compared the distribution of drought-associated DMPs with the genomic distribution of cytosine sites in each context. Across sequence contexts, DMPs were enriched in regions flanking genes (±1.5kb upstream and downstream) and depleted from intergenic regions (Figure 3A, Table S2), indicating that drought-responsive methylation changes preferentially occur near genes. Consistent with this pattern, DMPs occurred significantly closer to gene-flanking regions broadly, and upstream regions specifically, than expected from the genomic background in all contexts (upstream regions: Figure 3B; downstream regions: Figure S8). Context-specific differences emerged, however, in both genomic distribution and direction of methylation change. CG DMPs were depleted from TEs, while CHG DMPs were depleted from coding regions. In contrast, CHH DMPs were strongly enriched within and near TE sequences and remained enriched in gene-flanking regions (Figure 3A, Figure S9). This distribution mirrors both the baseline CHH methylation landscape observed in our data (Figure S5) and previous descriptions of plant methylomes (38, 39) in which CHH methylation is concentrated at gene-proximal TEs. Our results therefore indicate that drought preferentially remodels methylation within pre-existing regulatory regions rather than inducing methylation randomly across the genome. Moreover, whereas CG and CHG DMPs primarily represented losses of methylation, CHH DMPs were predominantly gains of methylation (Figure 2C). Consistent with this interpretation, the shift towards gene-proximal regions was strongest for CHH DMPs (Figure 3B and Figure S8), indicating that drought-responsive CHH hypermethylation preferentially targets TEs located near genes.

Associations between TEs and DMPs differed markedly among sequence contexts (Figure 3C). CHH DMPs showed strong enrichment within TEs, with 95% overlapping TE sequences compared with 68% of genomic CHH sites (fold enrichment = 1.4; χ^2^ = 78,331, adjusted *P* < 2.2 x 10−^16^). CHG DMPs were modestly depleted within TEs (62% versus 69%; fold enrichment = 0.89; χ^2^ = 4.97; adjusted *P* < 0.05), whereas CG DMPs were strongly depleted from TE regions relative to genomic expectations (43% versus 83%; fold enrichment = 0.52; χ^2^ = 353; adjusted *P* < 0.001). These patterns indicate that drought-responsive methylation was concentrated within TE-rich regions, primarily in the CHH context.

To determine whether drought-responsive CHH methylation was distributed uniformly across the TE landscape or preferentially targeted specific classes of elements, we examined enrichment across TE superfamilies. At broader taxonomic scales, CHH DMPs showed contrasting enrichment patterns across TE superfamilies (Figure 3D). Several DNA transposon and SINE superfamilies, including TcMar-Stowaway, MuDR, and tRNA-derived SINEs, were strongly enriched for CHH DMPs. At finer resolution, CHH methylation was highly structured at the level of individual TE families, with 312 families significantly enriched for TE-associated CHH DMPs (adjusted *P* < 0.05, Figure S10, Table S5). The fact that enrichment varied substantially among TE families within the same superfamily suggests that drought-responsive CHH methylation is associated with lineage-specific properties of individual TEs rather than broad TE classifications, consistent with selective targeting of TE families by DNA methylation machinery.

In a final test, we found that TE copies from enriched families showed feature-specific localization patterns. Specifically, TE copies were enriched in 1.5 kb upstream regions (odds ratio = 1.54) and, to a lesser extent, 1.5 kb downstream regions (odds ratio = 1.16), while gene bodies and introns were depleted (Figure 3E). Thus, drought-responsive CHH methylation is not randomly distributed among TEs but is concentrated in specific TE lineages positioned adjacent to genes, identifying gene-proximal TEs as candidate mediators of downstream gene expression.

### CHH-methylated upstream transposable elements are associated with drought-responsive gene expression

Drought-associated CHH hypermethylation at TEs was associated with altered expression of nearby genes. Genes bearing drought-induced CHH DMPs were more likely to have nearby TEs from CHH-DMP-enriched families than expected from the genomic background (generalized linear model: odds ratio = 4.16, *P* < 2.2 x 10−^16^), indicating that drought-responsive CHH methylation preferentially occurs at a subset of TE lineages with strong CHH-DMP enrichment. We therefore focused on these enriched TE families as candidates for mediating associations with drought-induced methylation and transcriptional responses.

**Figure 4.**
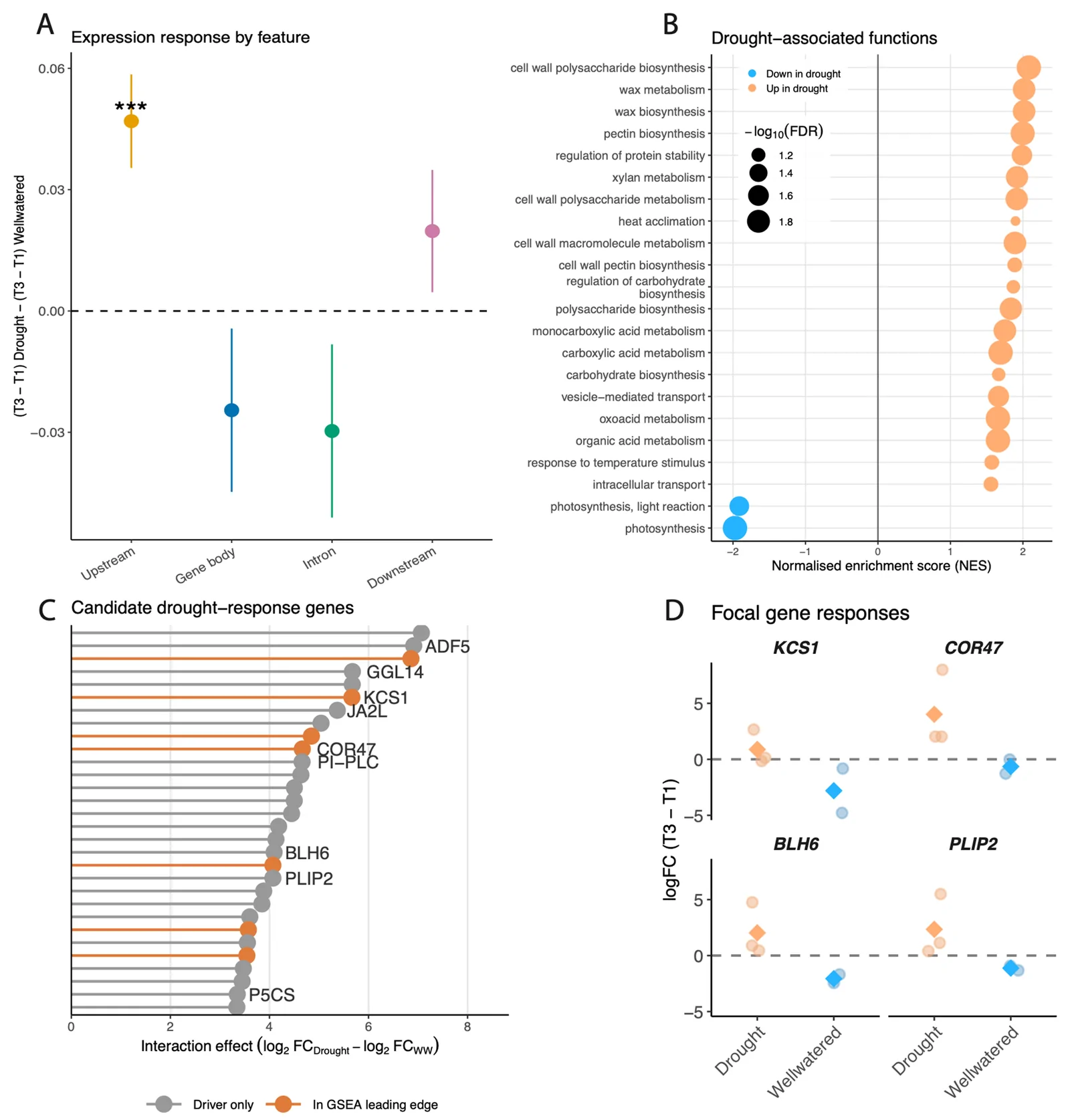
CHH methylation and expression dynamics of genes associated with transposable elements under drought. **(A)** Difference-in-differences in gene expression between drought-treated and well-watered seedlings for genes associated with CHH DMPs at four TE-linked feature classes: upstream (1.5kb), gene body, intron, and downstream (1.5kb). Values represent (T3-T1) _Drought_ – (T3- T1) _Well-watered._ Points show model estimates and error bars indicate standard errors from linear mixed-effects models. **(B)** Gene set enrichment analysis of genes ranked by their drought-versus-well-watered interaction effect. Enriched gene ontology (GO) biological process terms passing FDR < 0.10 are shown. Points are positioned by normalized enrichment score (NES), where positive values (orange) indicate enrichment among genes upregulated under drought and negative values (blue) indicate enrichment among genes downregulated under drought. Point size corresponds to −log10(FDR). Redundant GO terms describing overlapping carboxylic acid metabolic processes were removed for clarity. **(C)** Ranked interaction effects for top drought-responsive upstream TE-associated candidate genes. Genes were ordered by the difference in expression change between drought and well-watered seedlings, calculated as log2FCDrought– log2FCWell-watered. Each point represents a candidate gene associated with upstream TE-linked CHH methylation. Orange points indicate genes present in the leading edge of enriched GO terms in (B); labelled points indicate genes with strong evidence for a putative drought-response role (see Table S5 for full details). **(D)** Expression changes from T1 to T3 for four representative upstream TE-flanked genes carrying drought-associated CHH DMPs. The selected genes span distinct drought-response pathways: KCS1, wax biosynthesis; COR47, dehydrin-mediated stress protection; BLH6, transcriptional regulation; and PLIP2, lipid-derived stress signaling. Points show individual seedlings and diamonds indicate treatment means. Full gene details in Table S5.

Expression responses depended strongly on the position of methylated TEs relative to genes. Genes with CHH-hypermethylated TEs within 1.5kb upstream showed significantly higher transcription under drought than in well-watered controls, whereas no equivalent response was detected for gene body, intron, or downstream positions associated with TEs (Benjamini-Hochberg adjusted *P* = 2.0 x 10^−4^, Figure 4A). The upstream effect was specific to CHH methylation, with no significant expression response associated with CG nor CHG DMPs. A linear mixed-effects model supported this position-specific response, identifying a significant Treatment x Time x Position interaction (F_3,160020_ = 5.2, *P* = 0.0014), which remained significant when expression change was modelled directly as per-gene T3-T1 log_2_ fold change (Treatment x Feature: F_3,77043_ = 8.9, *P* = 6.7 × 10^−6^). Thus, contrary to the expectation that CHH methylation primarily represses adjacent genes, drought-induced CHH methylation at upstream TEs was associated with increased expression of nearby genes.

Gene set enrichment analysis identified a coherent set of genes activated under drought. Genes were ranked by their drought-versus-well-watered interaction effect and tested for enrichment of gene ontology (GO) biological process terms. Significant positively enriched terms included cell wall polysaccharide biosynthesis, pectin biosynthesis, xylan metabolism, wax biosynthesis, wax metabolism, carbohydrate biosynthesis, regulation of protein stability and heat acclimation (Figure 4B, Table S4). These functions are consistent with protective drought responses, including reinforcement and remodeling of the cell wall, modification of the cuticle, and stabilization of proteins under stress. In contrast, photosynthesis and light-reaction pathways were negatively enriched, consistent with drought-associated suppression of carbon assimilation and a physiological cost of stress exposure.

Candidate drought-response genes supported this functional pattern. Several highly ranked upstream TE-associated genes had known or putative roles in drought-related processes, including cuticle and wax metabolism (KCS1, GGL14) (41, 42), osmotic protection (COR47, P5CS) (43–45), phospholipid-derived stress signaling (PLIP2) (46), and transcriptional regulation (JA2L, BLH6) (47, 48), calcium or phosphoinositide-related signaling (PI-PLC) (49), and cytoskeletal remodeling (ADF5) (50) (Figure 4C, Table S5). Several of these genes also occurred in the leading-edge subsets of enriched GO categories, providing convergent support from both pathway- and gene-level analyses. Expression trajectories for representative genes confirmed increased expression under drought and stable or declining expression in well-watered controls (Figure 4D). These results indicate that drought-induced CHH methylation at upstream TEs is associated with activation of gene expression programs that may promote drought tolerance, while the concurrent downregulation of photosynthesis reflects the negative physiological impact of drought stress.

### Increasing CHH methylation is associated with attenuation of drought-responsive expression

**Figure 5.**
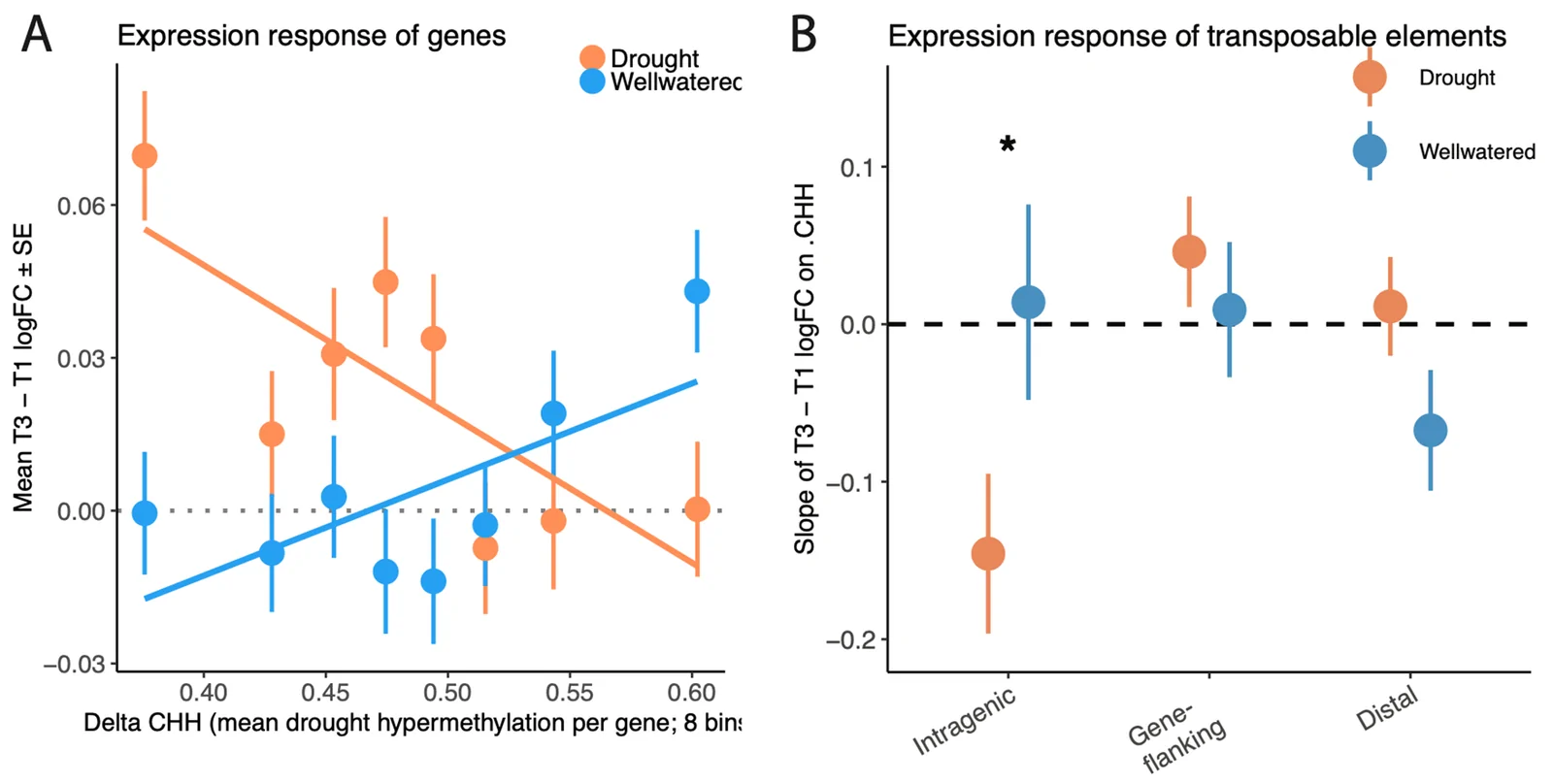
Dose-dependent relationship between CHH methylation, gene expression, and transposable element (TE) expression under drought. **(A)** Gene expression response as a function of CHH methylation change in genes associated with TE-linked CHH differentially methylated positions at all genomic positions: upstream (<1.5 kb), gene body, intron, and downstream (<1.5 kb). Points show mean ± SE gene expression change (T3 - T1 log_2_ fold change) across eight bins of increasing mean CHH methylation change (ΔCHH) per gene. Lines show fitted linear relationships from the mixed-effects model for drought-treated and well-watered seedlings. **(B)** Relationship between CHH methylation change and TE expression according to TE position relative to genes. Points show model estimated slopes (± SE) describing the relationship between ΔCHH and TE expression (log_2_ CPM) for drought-treated and well-watered seedlings at intragenic, gene-flanking (<1.5 kb from a gene), and distal (>1.5 kb from a gene) TE loci. Upstream and downstream TEs were combined into a single gene-flanking category because TE expression depends on TE position relative to genes rather than gene feature. Asterisks indicate a significant treatment difference in slope (* *P* < 0.05).

Genes associated with CHH methylation showed increased expression under drought (Figure 4), but the magnitude of this response declined with increasing CHH methylation. Genes carrying larger numbers of CHH DMPs at associated TEs showed significantly smaller expression gains under drought relative to well-watered controls (Treatment x DMP number: *F*_1,76835_ = 17.9, *P* = 2.3 × 10^−5^). This relationship was consistent across upstream, gene body, intronic, and downstream TE-associated classes (Position interaction: *P* = 0.23). A complementary model using the magnitude of methylation change (mean ΔCHH) produced the same result (Treatment x ΔCHH: *F*_1,77233_ = 8.8, *P* = 0.0029, Figure 5A). Across both analyses, increasing CHH methylation was associated with progressively weaker drought-induced transcriptional responses. These results suggest that the magnitude of drought-responsive expression is inversely related to local CHH methylation load.

To determine whether increasing CHH methylation was also associated with transcription of the methylated TEs themselves, we next analyzed TE transcription. Drought produced no detectable shift in TE expression at the superfamily level, indicating that short-term drought does not broadly activate or silence the TE transcriptome. However, the relationship between CHH hypermethylation and TE expression depended on genomic position (Treatment x DMP number x Position interaction: *P* = 0.037). This interaction was driven by intragenic TEs, where greater CHH methylation was associated with reduced TE expression under drought relative to well-watered controls (drought minus well-watered slope = −0.16 log_2_ CPM per unit ΔCHH, *P* = 0.045, Figure 5B). No comparable relationship was detected for upstream, downstream, or distal intergenic TEs (*P* > 0.05). Importantly, drought alone did not alter expression of intragenic TEs at average methylation levels (*P* = 0.58), indicating that repression scaled with the amount of CHH methylation acquired by individual loci rather than representing a uniform treatment effect. Intragenic TEs were also over-represented among both the most strongly up- and down-regulated loci (χ^2^ =22706, *P* < 2.2 x 10^−16^), suggesting that these elements occupy particularly dynamic transcriptional environments.

These results show that increasing levels of CHH methylation were associated with progressively weaker transcriptional responses of nearby genes and reduced expression of intragenic TEs. Thus, increasing CHH methylation was associated with attenuation of drought-responsive gene expression while maintaining repression of intragenic TEs.

## Discussion

Our study reveals three major processes that together provide new insight on how long-lived trees repeatedly regulate gene expression during environmental stress. First, repeated drought induces a reproducible DNA methylation response that repeatedly targets the same gene-proximal genomic regions despite turnover of individual methylated cytosines. Second, this genome-regulatory response is associated with gradients of expression in core drought-response pathways, including ABA signaling, osmotic adjustment, cuticle biosynthesis and cell-wall remodeling. Third, the same response is associated with continued repression of nearby transposable elements (TEs), suggesting that repeated stress coordinates adjustment of expression while maintaining local genome stability. Together, these results identify a reproducible genome-regulatory response associated with repeated environmental stress in a long-lived tree.

Repeated drought recruited a remarkably reproducible genome-regulatory response, despite extensive turnover of individual methylated cytosines. Although differential methylation occurred in all three sequence contexts, drought responses were overwhelmingly dominated by CHH hypermethylation concentrated within TEs and adjacent gene-flanking regions, with comparatively few CG or CHG changes. Notably, successive drought exposures repeatedly targeted the same gene-proximal TEs even though the precise methylated cytosines differed between stress events. This pattern indicates that repeated drought does not generate methylation randomly across the genome but instead repeatedly recruits RNA-directed DNA methylation (RdDM) to the same regulatory regions while allowing individual CHH marks to remain highly dynamic. Such regional reproducibility is consistent with the probabilistic targeting characteristic of the RdDM pathway (22, 23), suggesting that stress-responsive methylation is organized at the level of genomic neighborhoods rather than individual cytosines. We found no evidence that repeated drought physiologically primed seedlings, but the reproducible recruitment of methylation raises the possibility that the epigenomic response itself is repeatedly directed towards the same regulatory landscape. These regulatory regions were strongly enriched for gene-proximal TEs and specific TE lineages, consistent with growing evidence that TE identity, age and genomic position influence methylation dynamics and regulatory potential (51–55). Young TEs located near genes often retain greater epigenetic plasticity than deeply heterochromatic elements (51), potentially making them particularly responsive to environmental perturbation. Similar environmentally responsive CHH methylation has been described in Arabidopsis, rice, poplar and oak (20, 21, 34, 37), although both the magnitude and direction of methylation changes vary among systems, suggesting that CHH methylation represents a flexible regulatory response rather than a stereotyped feature of drought stress. Our findings extend these observations to a long-lived tree and demonstrate that repeated drought consistently recruits methylation to a reproducible set of gene-proximal regulatory regions, providing a framework for understanding how genome regulation is repeatedly deployed during environmental stress rather than simply documenting additional stress-induced CHH methylation.

The reproducible methylation pattern created by repeated drought was associated with gradients of drought-responsive expression, rather than simply activation or repression. Genes flanked by CHH methylated upstream TEs were enriched for pathways central to drought acclimation, including ABA signaling, osmotic stress tolerance, cuticle biosynthesis and cell-wall remodeling, indicating that these loci participate in the core transcriptional response to water limitation (4, 41, 43, 46, 56, 57). Pathway-level analyses similarly identified enrichment for cell-wall and wax biosynthesis, carbohydrate metabolism and ABA-responsive processes, whereas photosynthesis-related pathways were broadly downregulated, consistent with the well-established reallocation of resources from carbon acquisition towards stress tolerance during drought (4, 5). However, although genes adjacent to CHH-methylated TEs remained more highly expressed under drought overall, increasing CHH methylation was associated with progressively smaller transcriptional responses. Rather than functioning as a simple transcriptional on/ off switch, these results are consistent with an emerging view that environmentally responsive DNA methylation quantitatively modulates transcriptional output (26, 58–60). Experimental disruption of DNA methylation in Arabidopsis similarly revealed dose-dependent effects on both gene expression and TE repression (26), and our findings extend this concept to a long-lived tree by suggesting that environmentally responsive CHH methylation is associated with fine-scale tuning of stress-responsive expression during repeated environmental exposure.

One explanation for these gradients of expression is that maintaining local TE repression during periods of high gene activity carries consequences for neighboring genes. TE methylation is widely recognized as a mechanism that preserves genome integrity by suppressing TE activity (61–63) and Secco *et al.* (21) proposed that stress-induced CHH methylation may be particularly important in highly transcribed regions where nearby TEs become vulnerable to activation. A potential mechanistic basis for this relationship comes from Arabidopsis, where the SUVH1/SUVH3-DNAJ1/DNAJ2 methylation-reader complex binds RdDM-associated CHH methylation while promoting expression of nearby genes and maintaining TE silencing (25). Several observations from our study are consistent with a similar regulatory framework. First, repeated drought consistently recruited CHH methylation to the same gene-proximal TEs across independent stress events despite turnover of individual methylated cytosines. Second, drought produced little overall change in TE expression, while increasing CHH methylation within intragenic TEs was associated with reduced TE expression. Third, increasing CHH methylation was associated with progressively smaller expression responses in neighboring genes without abolishing expression altogether. Together, these observations are consistent with repeated drought recruiting methylation to specific gene-proximal regulatory regions where genome stability is maintained while the magnitude of nearby stress-responsive transcription is modulated. Our results support the hypothesis that environmentally responsive CHH methylation contributes to balancing robust transcriptional responses with continued repression of nearby TEs, although they cannot establish causality. Whether these gradients ultimately enhance stress resilience of instead reflects a trade-off associated with maintaining genome stability during repeated environmental stress remains an important question for future functional studies in long-lived trees.

The molecular basis of this dose-dependent response remains unresolved but the findings from our study provide important clues about how drought-responsive methylation is generated. Despite widespread CHH gains, core RdDM genes showed little transcriptional response (Figure S11, Table S6). Only HEN1, which stabilizes small RNAs through 3’ terminal methylation (62, 64, 65), was significantly up-regulated, suggesting that drought may act primarily through altered small RNA dynamics and targeting. This up-regulation might explain how extensive CHH gains can occur without activation of core RdDM enzymes, because methylation targeting is determined largely by siRNA production and recruitment rather than enzyme activity alone (62, 66).

In conclusion, our findings identify a specific mechanism that plays a role in the adjustment of trees to repeated environmental stress: a reproducible genome-regulatory response that facilitates this adjustment. While environmentally responsive DNA methylation has been described in numerous plant species, to our knowledge this is the first study in a tree to show that repeated drought repeatedly induces DNA methylation to the same regulatory regions despite continual turnover of individual methylated cytosines. By linking this reproducible response to the magnitude of drought-responsive gene expression, our study suggests that stress-responsive genome regulation operates at the level of regulatory regions rather than individual methylated sites. These findings provide a framework for understanding how long-lived trees repeatedly adjust gene expression while maintaining genome stability during recurring environmental stress over decades to centuries, and future functional studies will be required to establish causality in this proposed mechanism.

## Methods

### Sample design and drought assays

In October 2022, acorns were sampled from seven *Q. lobata* trees growing in oak savannah habitat growing in Sedgwick Reserve, located in the Santa Ynez Valley, Santa Barbara County, CA, (Figure S1) and administered by the University of California Santa Barbara, as part of the University of California’s natural reserve system.

To assess the physiological responses to the soil drying and well-watered treatments, we monitored the progress of all seedlings from each maternal tree (Tree IDs were 530, 667, 27, 165, 786, 186, and 896). Acorns were surface-sterilized and planted in a randomized design in the greenhouse at UCLA. In July 2023, after approximately seven months since germination, seedlings from each maternal family (i.e., half-siblings from seven maternal trees) were randomly assigned to drought and well-watered treatment groups. Twice weekly, seedling transpiration was measured using a Licor Li-600 porometer and soil moisture measured using a Hobo MX2307 probe to monitor seedling response to drought. Three readings were taken per seedling from the newest fully expanded leaves. Well-watered seedlings were watered every few days, while drought seedlings underwent a first drought period for ten days, after which they were watered until their stomatal conductance rate returned to the same level as the well-watered seedlings. A second and longer drought period for twenty days was commenced. We followed methods developed in previous drought studies completed (67–69).

For the genome-wide methylation profiling and gene expression studies, we selected eight half-sib seedlings from two maternal families (667 and 786), comprising two seedlings per family in each treatment (drought and well-watered). Leaf samples were collected before treatment application (timepoint 1, T1); at the end of the first ten-day treatment (timepoint 2, T2); and ten days into the second drought treatment following rewatering (timepoint 3, T3). Each seedling was sampled across all three timepoints, drought responses to be evaluated relative to two complementary controls: (i) contemporaneous well-watered seedlings from the same maternal families; and (ii) pre-treatment measurements from the same individual seedlings. This repeated-measures design enabled drought-associated responses to be distinguished from both baseline inter-individual variation and temporal changes unrelated to drought.

### Oxford Nanopore PromethION genome and methylome sequencing

High molecular weight genomic DNA was extracted from half of each young leaf sample following the cetyltrimethylammonium bromide (CTAB) method described by (70), with the following modifications: (i) sodium metabisulfite (1% w/v) was used instead of 2-mercaptoethanol (1% v/v) in the sorbitol wash buffer and CTAB solution; (ii) tissue homogenate wash steps were repeated until the supernatant became clear; (iii) the CTAB lysis step was performed at 45°C; and (iv) the chloroform extraction step was performed twice using ice-cold chloroform. DNA purity was assessed by absorbance ratios measured using the NanoDrop ND-1000 spectrophotometer (Thermo Fisher Scientific, MA). DNA yield was quantified using the QuantiFluor ONE dsDNA Dye assay (Promega, WI). The size distribution of the high molecular weight DNA was estimated using the Femto Pulse system with the Genomic DNA 165 kb kit (Agilent, CA).

Libraries were sequenced at University of California Davis DNA Technologies core facility (Davis, CA) using a PromethION flowcell for 48 h. Nucleotide bases and all-context methylation were called from the raw sequence data using an A5000 GPU box with Dorado 0.5.0, after which sequencing data was aligned to the valley oak reference genome (accession GCF 001633185.2) during modified base calling. Methylation data in CG, CHG, and CHH sequence contexts were extracted from the resulting bam files using modkit v0.2.8 with “modkit pileup --ignore h” parameters. The raw sequence data has been deposited in the International Nucleotide Sequence Database Collaboration (INSDC) database under the BioProject accession number XXXXXXX.

### Differentially methylated positions

Differential methylation was assessed independently for each individual seedling, sequence context, and timepoint comparison (T1-T2 and T1-T3) using modkit v0.2.8 “dmr pair --base C”. Analyses were conducted at single-cytosine resolution and generated per-site estimates of methylation change, methylation frequency and coverage for each comparison. Candidate differentially methylated positions (DMPs) were identified at *P* < 0.05 and required a minimum coverage of five reads at both timepoints. Subsequent catalogue construction required reproducibility across independent seedling replicates and agreement in the direction of methylation change, providing a substantially more stringent filter than the per-site significance threshold alone. Because drought responses were quantified relative to each seedling’s pre-treatment methylation state, inference was based on within-individual temporal change rather than solely on between-treatment comparisons. Drought-associated DMPs therefore represent loci showing consistent temporal responses across seedling replicates while also differing from changes observed in well-watered controls. Specifically, to identify reproducible drought-associated methylation changes, candidate DMPs were compared across drought-treated individuals using a single-base overlap with a ±10 bp tolerance. DMPs were required to be present in at least three of four individuals and show concordant directionality of methylation change across individuals (Supplementary Methods).

An independent control DMP catalogue was generated using the same workflow applied to drought-treated individuals, including coverage filtering, concordance requirements and direction matching. This catalogue was used as a reference for control masking during drought DMP identification. Drought-associated DMPs were defined relative using a difference-in-differences framework, whereby methylation changes through time in drought-treated seedlings were evaluated relative to contemporaneous changes observed in well-watered controls. This approach follows the same treatment x time logic commonly used to estimate treatment-specific responses in longitudinal transcriptomic studies (71). For each candidate locus, the drought effect size was compared with the mean effect size observed in controls, and loci were retained when divergence from the control response exceeded a threshold of δ = 0.10 in the drought direction.

To assess whether drought-responsive methylation persisted across successive drought exposures, DMP catalogues generated for the initial drought (T1-T2) and repeated drought (T1-T3) were compared separately for each sequence context (CG, CHG, and CHH). The same individual (“seed” individual) was used to anchor the construction of a concordance-based DMP catalogue at both timepoints, so genomic positions could be compared directly between contrasts. Shared DMPs were defined as loci occurring within ±10 bp and exhibiting the same direction of methylation change (hyper- or hypomethylation). For each sequence context, the proportion of T1-T2 DMPs that recurred at T1-T3 was calculated as the DMP memory rate. To determine whether repeated drought targeted the same regions despite turnover of individual methylated cytosines, DMP was overlap was also assessed at the level of TEs. The proportion of TEs containing CHH DMPs in both drought contrasts was compared with a null expectation generated by randomly sampling callable CHH cytosines from the genome while preserving the observed number of DMPs. Enrichment relative to the null distribution was estimated from 1000 permutations. This analysis was repeated using each drought-treated individual as the catalogue seed. Positional overlap was recalculated across a series of matching windows ranging from exact cytosine matches (0 bp) to ±500 bp, allowing assessment of whether repeated drought targeted individual cytosines or nearby cytosines within the same genomic regions.

### Genomic annotation and enrichment analyses

DMPs were annotated relative to the gene models from the valley oak 3.2 genome annotation (accession GCF 001633185.2). Strand-aware genomic features were defined as upstream (1.5 kb), gene body, intronic, and downstream (1.5 kb) regions. Transposable elements were identified using the RepeatMasker annotation of the valley oak assembly. RepeatModeler rnd-N_family-M identifiers were further grouped into s1RF family clusters using a 45% sequence-identity threshold for family-level analyses. DMPs were assigned to gene features by interval overlap. Associations with CHH methylation and TEs were assessed using a 269-bp proximity threshold, corresponding to the 90^th^ percentile of observed DMP-TE distances (Figure S9). Distances between DMPs and the nearest gene feature or TE were calculated using “bedtools closest.” Enrichment of DMPs within gene features was evaluated separately for each sequence context using Fisher’s exact tests against a background of covered cytosines. The cytosine background was restricted to positions with valid coverage ≥5x at both timepoints in all eight individuals, matching the depth threshold required for DMP calling. Enrichment of CHH DMPs among TE superfamilies and s1RF families was assessed for TE categories exceeding 10 kb of genomic coverage.

For each sequence context, DMPs were classified as gains or losses of methylation and summarized across gene feature classes. Associations between methylation direction and feature classes were assessed using χ^2^ tests.

### RNA extraction, sequencing, and orthology assignment

Gene expression was quantified in *Q. lobata* seedlings from the maternal families of Tree #s 667 and 786 exposed to drought and well-watered treatments. Expression analyses were conducted to evaluate associations between drought-induced CHH methylation and transcriptional responses. Because the primary methylation analyses were based on within-individual changes between T1 and T3, expression analyses were likewise restricted to paired T1-T3 comparisons, allowing each seedling to serve as its own baseline control. As in the methylation analyses, contemporaneous well-watered controls were retained throughout, enabling drought-induced expression changes to be distinguished from temporal changes unrelated to drought. RNA-seq libraries from several seedlings failed quality-control filtering, reducing the number of individuals available for analysis. We therefore retained the five individuals with complete paired T1 and T3 transcriptomes (three drought and two well-watered). These individuals were drawn from the same experiment and maternal families as those used for methylation profiling, although the RNA-seq and methylation datasets were only partially overlapping owing to sample-specific quality-control filtering.

Leaf tissue was frozen in liquid nitrogen and ground with beads. To extract RNA, we added 1.8 mL cold extraction buffer to each tube consisting of 8 M urea, 3 M LiCl, 1.76% polyvinylpyrrolidone K-60 solution, and 10 mM dithiothreitol (added from 1 M stock just before use). Tubes were vortexed vigorously and ground for 10 secs at 30 Hz, followed by centrifugation for 10 min at 1,000 rcf and 4° C that allowed recovery of 1.4 mL supernatant, which was kept at 4° C for twenty minutes. Next, we centrifuged tubes for 30 min at 20,000 rcf and 4 °C. After discarding the supernatant, the pellet was washed with 2 rounds of 70% ethanol, each followed by a 5-min centrifugation at 5,000 rcf and room temperature. Finally, the pellet was dried in the fume hood for 10 min. The air-dried pellet was used as the starting material for extraction of total RNA with the RNeasy Plant Mini Kit (QIAGEN, Germantown, MD). RNA was quantified using a NanoDrop 2000 (Thermo Fisher), with the RNA quality assessment (Qubit and Fragment Analyzer), library preparation, and Next Generation Sequencing performed by DNA Technologies and Expression Analysis Core at the UC Davis Genome Center.

Libraries were prepared using 3’ Tag-Seq and sequenced to a depth of 3-6 million single-end reads per sample. Because 3’ Tag-Seq relies on oligo-dT priming, expression estimates reflect polyadenylated transcripts and do not capture small RNAs associated with RNA-directed DNA methylation.

Raw sequenced reads were quality- and adapter trimmed using Trimmomatic v0.39 (parameters: ILLUMINACLIP: $TRIMMOMATIC DIR/adapters/TruSeq3PE.fa:2:30:10 SLIDINGWINDOW:4:5 LEADING:5 TRAILING:5 MINLEN:25) (72). Reads were quality checked with FASTQC v0.11.2 (73). To remove ribosomal RNA sequences, reads were mapped to the SILVA rRNA database (74) using BBTools “bbmap” with the parameter “outu” to keep only the unmapped reads. Reads were aligned to the valley oak 3.2 reference genome (accession GCF 001633185.2) using STAR v2.7.11b. Multi-mapping reads were retained with a maximum of 100 alignments per read to avoid systematically excluding transcripts originating from repetitive genomic regions. CPM-normalized coverage tracks were generated using “deepTools bamCoverage” and gene-level expression was quantified by summing CPM coverage across annotated gene bodies using “bedtools map.” Expression values were analyzed as log_2_(CPM + 1).

For functional analyses, oak genes were assigned to *Arabidopsis thaliana* orthologs using the best SwissProt hit from the Trinotate annotation. Arabidopsis identifiers were resolved to TAIR loci using UniProt-TAIR mappings, retaining the highest-confidence hit for each oak gene.

### Expression responses associated with CHH methylation

Analyses focused primarily on CHH methylation because drought-induced methylation responses were dominated by this sequence context and were restricted to genes associated with CHH DMPs overlapping TE families that were significantly enriched for CHH methylation. Gene-expression responses were analyzed using linear mixed-effects models implemented in lmerTest (75). The primary model included treatment, sampling timepoint and DMP feature class (upstream, gene body, intronic and downstream) as fixed effects and gene identity and individual identity as random intercepts. Difference-in-differences contrasts were extracted using emmeans (76). Multiple testing correction was performed using the Benjamini-Hochberg procedure and reported as FDR. Additional models examined whether transcriptional responses scaled with either the number of nearby CHH DMPs or the mean magnitude of local CHH methylation change. Marginal slopes and treatment-specific trends were estimated using emtrends. Genes were ranked according to their treatment-specific expression response, calculated as the difference between drought and well-watered log-fold changes. Gene-set enrichment analysis (GSEA) was performed using clusterProfiler (77) with the biological-process ontology and Arabidopsis ortholog assignments.

### Transposable element expression analyses

Expression of reference TEs was quantified using the RepeatMasker annotation of the valley oak 3.2 genome. CPM-normalized coverage was summed across annotated TE coordinates, and expression responses were calculated as T3-T1 log-fold changes. TE-expression responses were analyzed using linear mixed-effects models with TE superfamily as a fixed effect and individual identity as a random effect. Treatment-specific contrasts were estimated using emmeans. To test whether TE-expression responses were associated with local methylation changes, CHH DMP counts were intersected with TE coordinates and incorporated into mixed-effects models. TEs were additionally classified as intragenic, gene-flanking (<1.5 kb from the nearest gene) or distal. Treatment-specific methylation-expression relationships were estimated using emtrends.

### Methylation machinery candidate genes

Expression of candidate DNA methylation machinery genes was examined. Candidate genes included maintenance and de novo methyltransferases, demethylases, RNA-directed DNA methylation components and associated chromatin regulators. Arabidopsis reference genes were mapped to oak homologues using the same orthology-assignment procedure described above. For each candidate gene, treatment effects were evaluated using drought-versus-control differences in T3-T1 expression change.

## Supplementary Material

1

## Data Availability

Statistical analyses and visualization were performed in R 4.4.0 (78) using the following packages: lmerTest, emmeans, emtrends, clusterProfiler. All analysis and plotting codes are published at https://github.com/lilypeck/oak-drought-methylation.

## Significance statement

Plants cannot escape environmental change, and trees must repeatedly respond to stresses, such as drought, over lifetimes spanning decades to centuries. Yet little is known about the molecular mechanisms that make this remarkable resilience possible. Using a widespread California oak, we show that repeated drought repeatedly induced the same DNA methylation pattern in the same parts of the genome, even though the differentially methylated individual sites changed between drought events. This pattern was linked to how strongly drought-response genes were activated, suggesting that trees repeatedly deploy the same molecular program to respond to environmental stress. Our findings provide a new framework for understanding how long-lived organisms repeatedly adjust to changing climates.
