# Supplementary material for: Repeated drought induces a reproducible DNA methylation response associated with gene expression in Quercus lobata
Source: bioRxiv. 2026 Sep 7:2026.07.14.738593. Originally published 2026 Jul 20. Preprint. [Version 2] doi: 10.64898/2026.07.14.738593 (PMC13419498; doi:10.64898/2026.07.14.738593)
Supplement: Supplementary file 1 [file NIHPP2026.07.14.738593v2-supplement-1.pdf]

bioRxiv preprint doi: https://doi.org/10.64898/2026.07.14.738593; this version posted September 7, 2026. The copyright holder for this preprint (which was not certified by peer review) is the author/funder, who has granted bioRxiv a license to display the preprint in perpetuity. It is made available under a CC-BY 4.0 International license.

Supplementary Materials for

Repeated drought induces a reproducible DNA methylation response associated with gene expression in *Quercus lobata*

Lily D. Peck* and Victoria L. Sork*

*Corresponding author. Email: ldpeck@ucla.edu; vlsork@ucla.edu

This PDF file includes:
Supplementary Text
Figs. S1 to S11
Tables S1 to S6

bioRxiv preprint doi: https://doi.org/10.64898/2026.07.14.738593; this version posted September 7, 2026. The copyright holder for this preprint (which was not certified by peer review) is the author/funder, who has granted bioRxiv a license to display the preprint in perpetuity. It is made available under a CC-BY 4.0 International license.

Supplementary Text

Sensitivity analyses

For the stable maintenance contexts CG and CHG, we recovered the same number of DMPs whether we required each position to be present in 3/4 versus 4/4 individuals. Whereas for the CHH context, which is substantially noisier on a per-individual basis, full 4/4 concordance discards genuine events to per-individual stochasticity; we therefore relaxed the CHH threshold to 3 of 4 individuals ("relaxed"). This asymmetry was validated empirically: relative to the strict 4/4 CHH catalogue, the relaxed 3/4 catalogue recovered roughly 16-fold more CHH DMPs while preserving the same biological signatures, namely the fraction overlapping annotated transposable elements (90.0% strict versus 90.6% relaxed) and the per-site effect-size distribution (median ΔCHH 0.46 versus 0.48) were essentially identical, and the directional composition was unchanged (~99.8% hypermethylated in both). The relaxation therefore recovers real CHH methylation events rather than admitting noise.

bioRxiv preprint doi: https://doi.org/10.64898/2026.07.14.738593; this version posted September 7, 2026. The copyright holder for this preprint (which was not certified by peer review) is the author/funder, who has granted bioRxiv a license to display the preprint in perpetuity. It is made available under a CC-BY 4.0 International license.

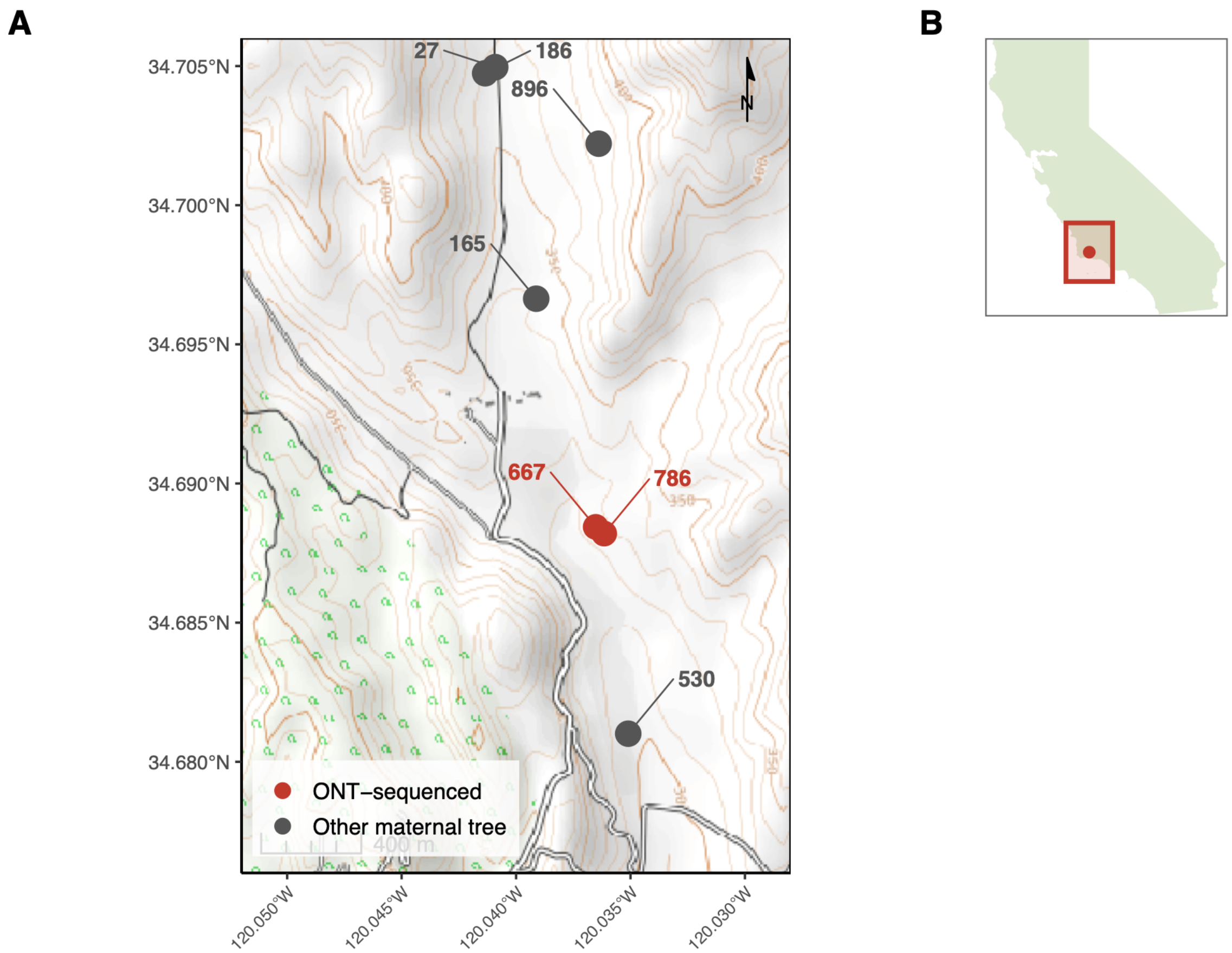

C

Individual	Family	Treatment	Mean depth T1	Mean depth T2	Mean depth T3	Mean background CG (M)	Mean background CHG (M)	Mean background CHH (M)
667_C1	667	Control	16.4	16.4	23.3	21.8	31.0	207.1
667_C2	667	Control	20.6	22.3	19.3	21.8	30.9	206.7
667_D1	667	Drought	11.8	28.1	14.3	21.6	30.6	205.1
667_D2	667	Drought	17.3	13.3	17.7	21.7	30.8	206.4
786_C1	786	Control	23.6	19.1	20.4	23.3	32.7	217.1
786_C3	786	Control	25.5	18.6	14.9	23.3	32.7	217.5
786_D2	786	Drought	22.7	19.3	15.6	23.2	32.5	216.2
786_D3	786	Drought	16.00	14.3	22.5	23.2	32.5	216.1

Figure S1. Study site and sequenced individuals. (A) Map of the locations of seven *Quercus lobata* trees sampled for acorn collection at the UC Santa Barbara Sedgwick Reserve, Santa Barbara County, California. Trees from two maternal families (667 and 786; red circles) were selected for Oxford Nanopore sequencing; remaining maternal trees (black circles) contributed seedlings to the drought phenotyping experiment only. Basemap: OpenTopoMap. (B) Location of Sedgwick Reserve within map of California. (C) Oxford Nanopore sequencing metrics for eight seedlings collected from the 667 and 786 maternal trees and which were included in methylation analysis (four per maternal family × two treatments). Mean depth represents the average read depth across cytosine sites for

bioRxiv preprint doi: https://doi.org/10.64898/2026.07.14.738593; this version posted September 7, 2026. The copyright holder for this preprint (which was not certified by peer review) is the author/funder, who has granted bioRxiv a license to display the preprint in perpetuity. It is made available under a CC-BY 4.0 International license.

the CG context (CHG and CHH values are similar). Mean background represents site counts averaged across T1–T3 comparisons. M = million cytosine sites.

bioRxiv preprint doi: https://doi.org/10.64898/2026.07.14.738593; this version posted September 7, 2026. The copyright holder for this preprint (which was not certified by peer review) is the author/funder, who has granted bioRxiv a license to display the preprint in perpetuity. It is made available under a CC-BY 4.0 International license.

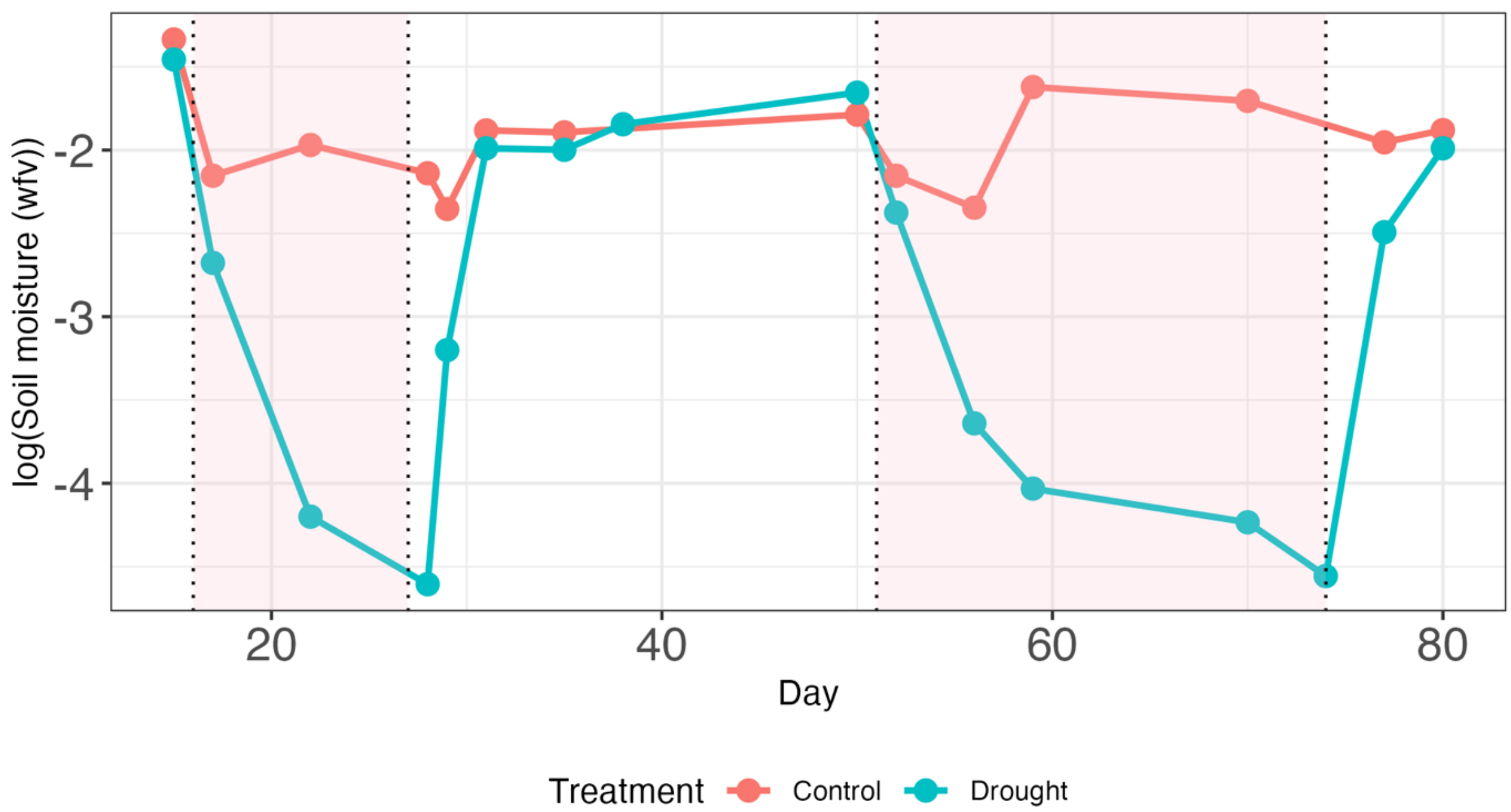

Figure S2. Measurements of soil water content during the experiment. Time course of soil moisture (water fraction by volume) during the experiment for the well-watered and drought stress treatments. Water deficit started on day 0 (T1) and lasted for 10 days (T2), after which plants were rewatered until ecophysiology measurements returned to similar values as in the well-watered treatment. A second drought was then initiated, for 20 days with plants measured and sampled after 10 days (T3).

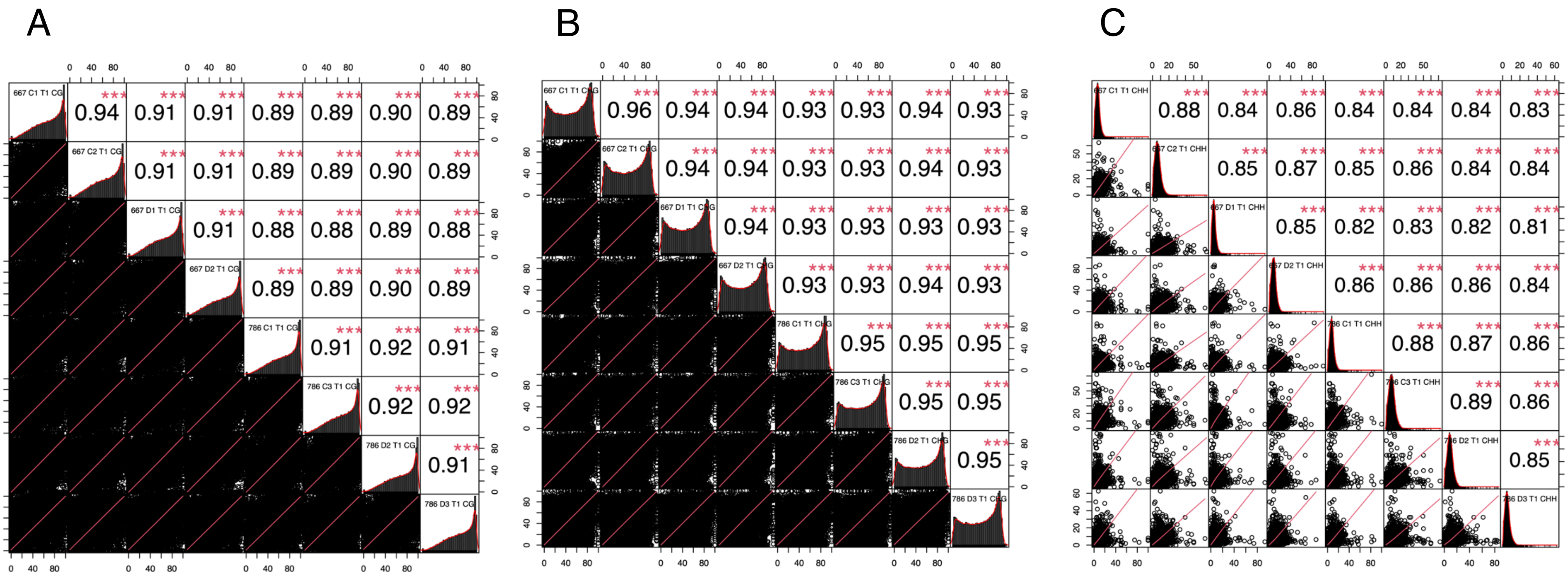

Figure S3. DNA methylation correlation analysis among all seedling replicates at timepoint 1. Methylation levels were calculated for each 10 kb bin along chromosomes, and Pearson correlation coefficient and significance was estimated for each bin between all individual seedlings at timepoint 1, before the onset of treatment. A Correlations in the CG context B Correlations in the CHG context C Correlations in the CHH context.

bioRxiv preprint doi: https://doi.org/10.64898/2026.07.14.738593; this version posted September 7, 2026. The copyright holder for this preprint (which was not certified by peer review) is the author/funder, who has granted bioRxiv a license to display the preprint in perpetuity. It is made available under a CC-BY 4.0 International license.

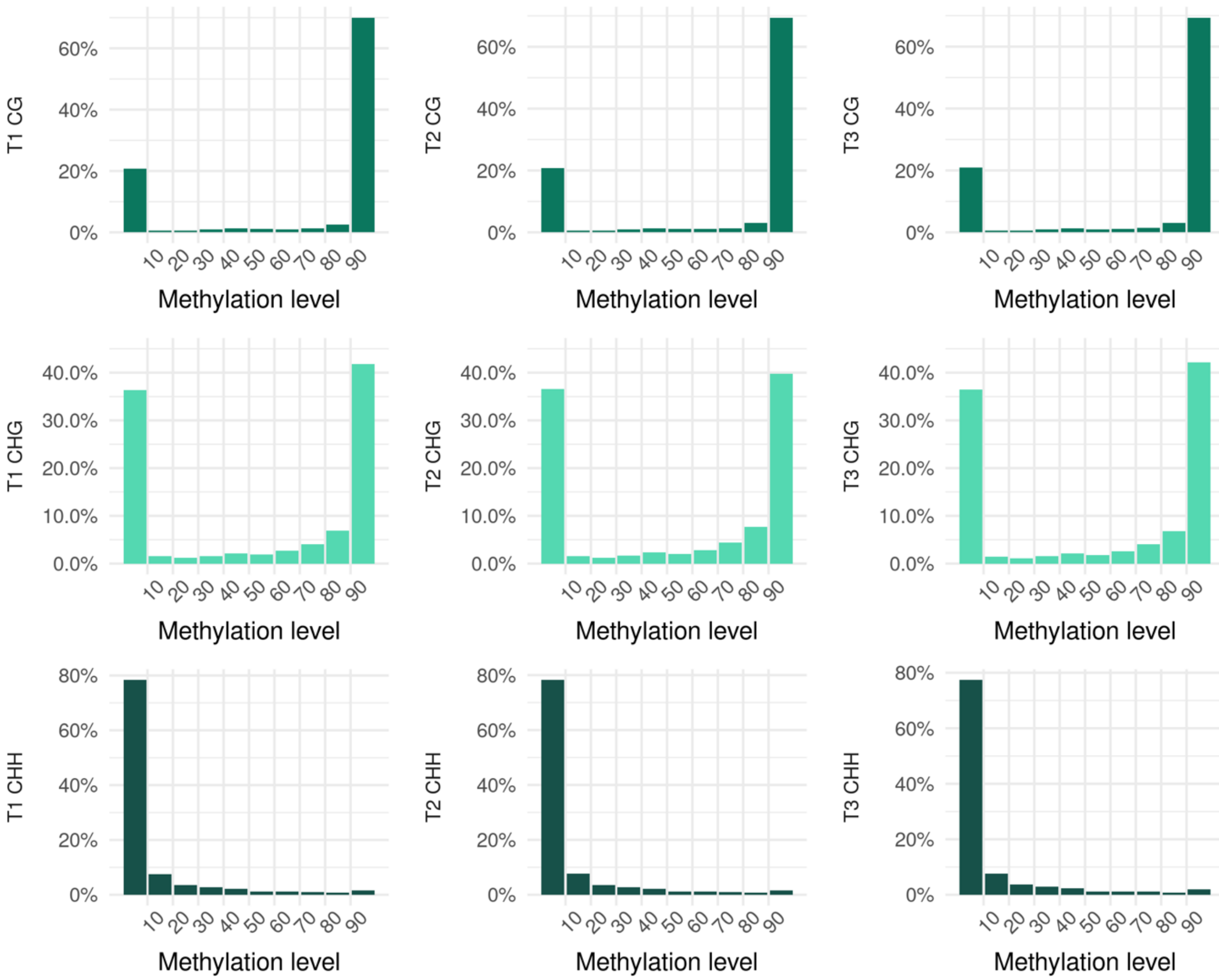

Figure S4. DNA methylation level distribution of CG, CHG and CHH at each timepoint. Data is shown for individual two of the maternal family 786 drought treatment as a representative sample of all individuals. The methylation levels were divided into 10 bins from 0 to 100%.

bioRxiv preprint doi: https://doi.org/10.64898/2026.07.14.738593; this version posted September 7, 2026. The copyright holder for this preprint (which was not certified by peer review) is the author/funder, who has granted bioRxiv a license to display the preprint in perpetuity. It is made available under a CC-BY 4.0 International license.

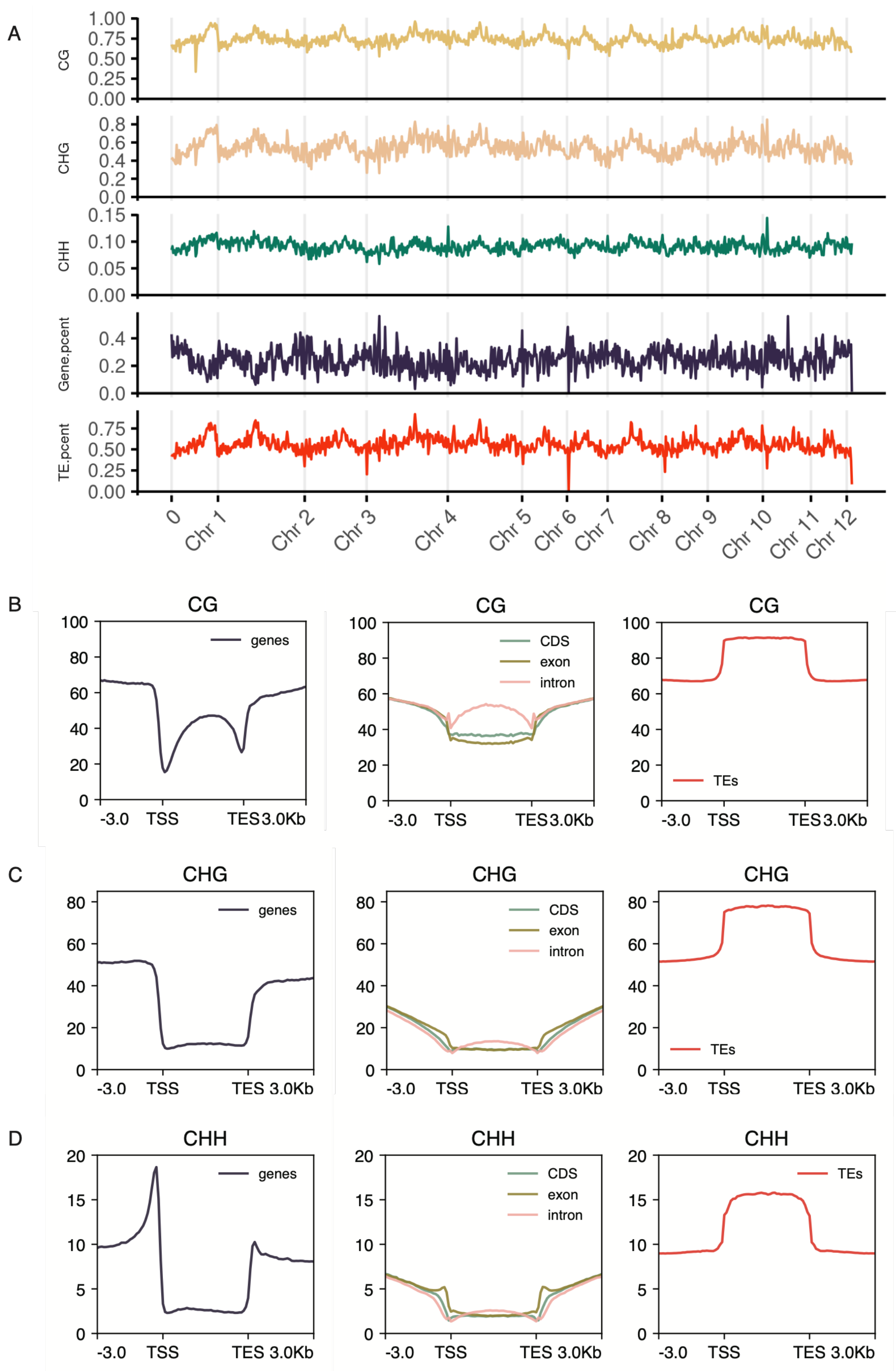

Figure S5. Characterisation of the DNA methylome of *Quercus lobata.* A Methylation, gene and transposable element (TE) features depicted by using 1Mb-wide bins across all 12 chromosomes, shown with grey vertical lines, at T1 (pre-drought) in the individual 786-D2. Y-axis shows proportions of methylation (CG, coloured yellow, top bar; CHG, coloured peach, second bar; CHH, coloured turquoise, third bar), genes (coloured purple, fourth bar), and TEs (coloured red, bottom bar) across each 1Mb bin calculated using 'bedtools map' with no sliding bins. B-D DNA methylation levels for genes, coding and non-coding regions, and TEs for CG (B), CHG (C), and CHH (D) methylation.

bioRxiv preprint doi: https://doi.org/10.64898/2026.07.14.738593; this version posted September 7, 2026. The copyright holder for this preprint (which was not certified by peer review) is the author/funder, who has granted bioRxiv a license to display the preprint in perpetuity. It is made available under a CC-BY 4.0 International license.

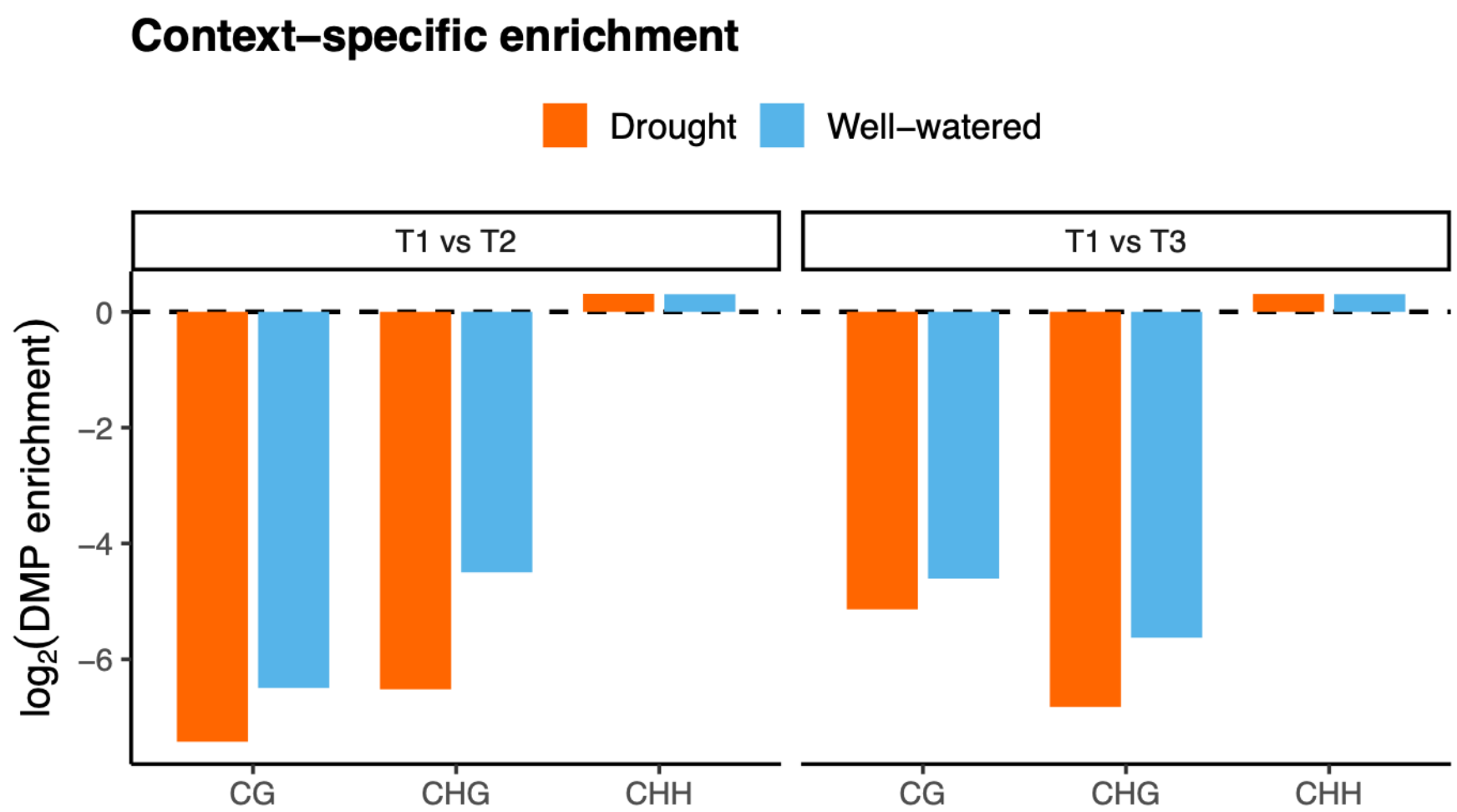

Figure S6. Enrichment of drought-associated differentially methylated positions (DMPs) across sequence contexts relative to genomic abundance. Bars show $\log_2$-transformed observed-to-expected proportions of DMPs relative to the number of genomic cytosine positions, namely the genomic background, for each sequence context. Significance of enrichment or depletion was assessed using chi-square tests comparing observed DMP frequencies to background cytosine frequencies within each context. For each sequence context and timepoint, the observed number of DMPs differed significantly from the genomic cytosine background (BH-adjusted $P < 0.001$), with CHH significantly enriched and CG and CHG depleted.

bioRxiv preprint doi: https://doi.org/10.64898/2026.07.14.738593; this version posted September 7, 2026. The copyright holder for this preprint (which was not certified by peer review) is the author/funder, who has granted bioRxiv a license to display the preprint in perpetuity. It is made available under a CC-BY 4.0 International license.

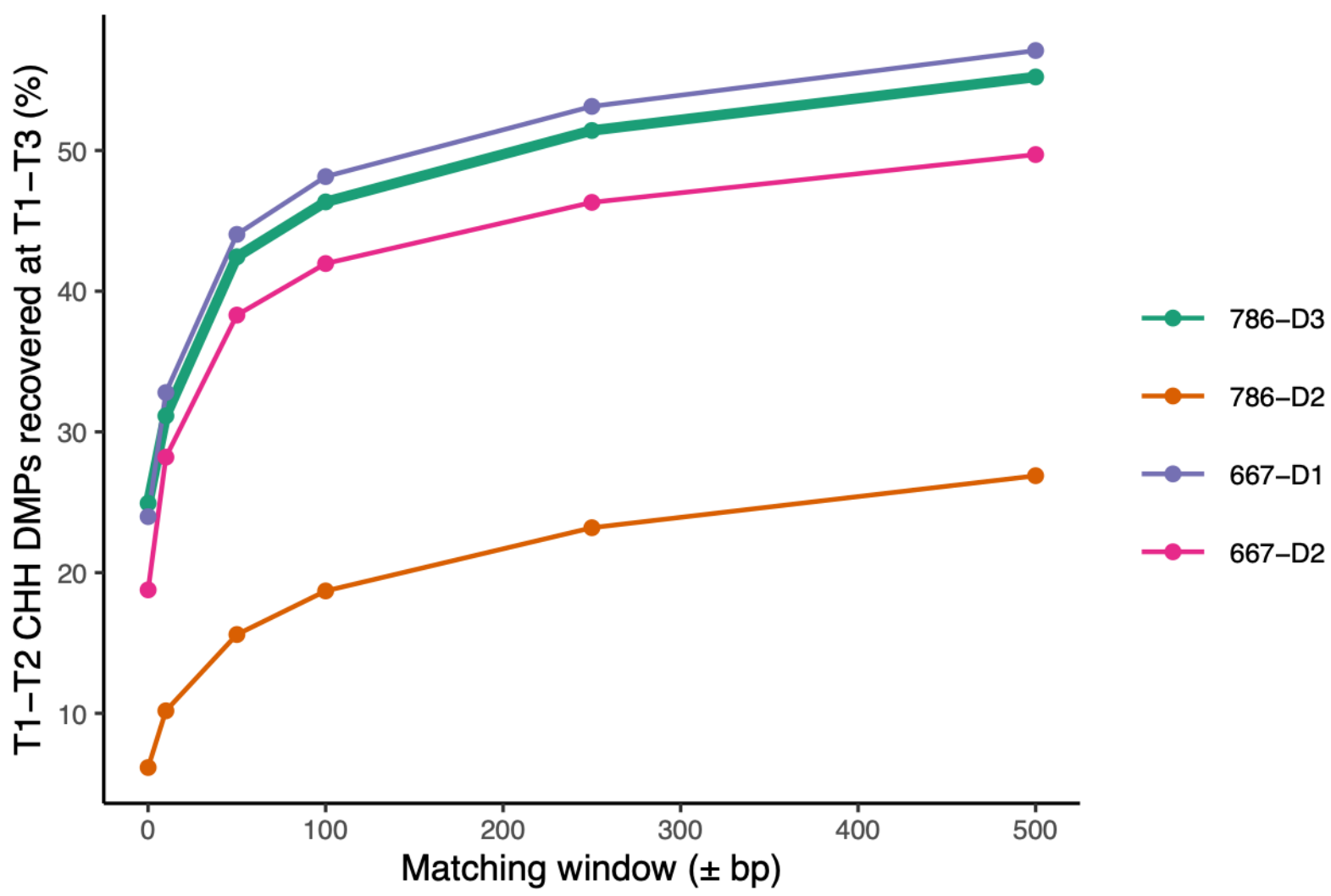

Figure S7. The number of CHH differentially methylated positions (DMPs) that recurred between T1-T2 and T1-T3 among individuals. CHH DMP recurrence between the first (T1–T2) and second (T1–T3) drought exposures was recalculated using each of the four drought-treated seedlings as the seed for the DMP catalogue. The y-axis shows the percentage of T1–T2 CHH DMPs recovered at T1–T3, while the x-axis shows the positional matching window used to define recurrent (0 bp = exact cytosine match). Similar curves across seed individuals demonstrate that the observed recurrence pattern was robust to catalogue construction.

bioRxiv preprint doi: https://doi.org/10.64898/2026.07.14.738593; this version posted September 7, 2026. The copyright holder for this preprint (which was not certified by peer review) is the author/funder, who has granted bioRxiv a license to display the preprint in perpetuity. It is made available under a CC-BY 4.0 International license.

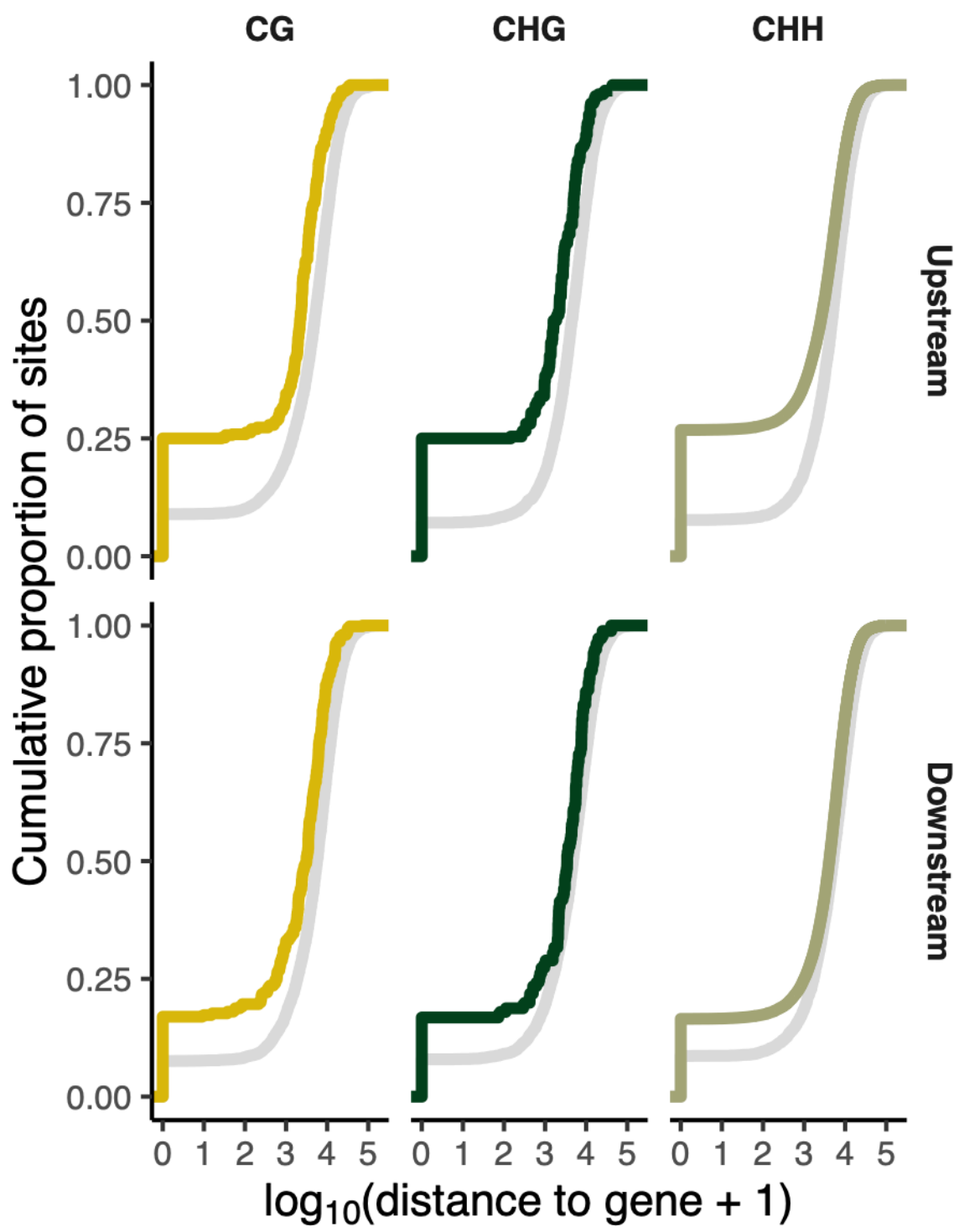

Figure S8. Drought-associated differentially methylated positions (DMPs) occur closer to gene-flanking regions than expected from the genomic cytosine background. Cumulative distributions show the distance from each drought-associated DMP (coloured) or genomic cytosine background (grey) to the nearest upstream (top) or downstream (bottom) gene-flanking region (±1.5 kb). Distances are plotted as log10(distance + 1). The coloured curves accumulate more rapidly than the genomic background, indicating that DMPs occur significantly closer to gene-flanking regions than expected, with the strongest enrichment observed upstream of genes.

bioRxiv preprint doi: https://doi.org/10.64898/2026.07.14.738593; this version posted September 7, 2026. The copyright holder for this preprint (which was not certified by peer review) is the author/funder, who has granted bioRxiv a license to display the preprint in perpetuity. It is made available under a CC-BY 4.0 International license.

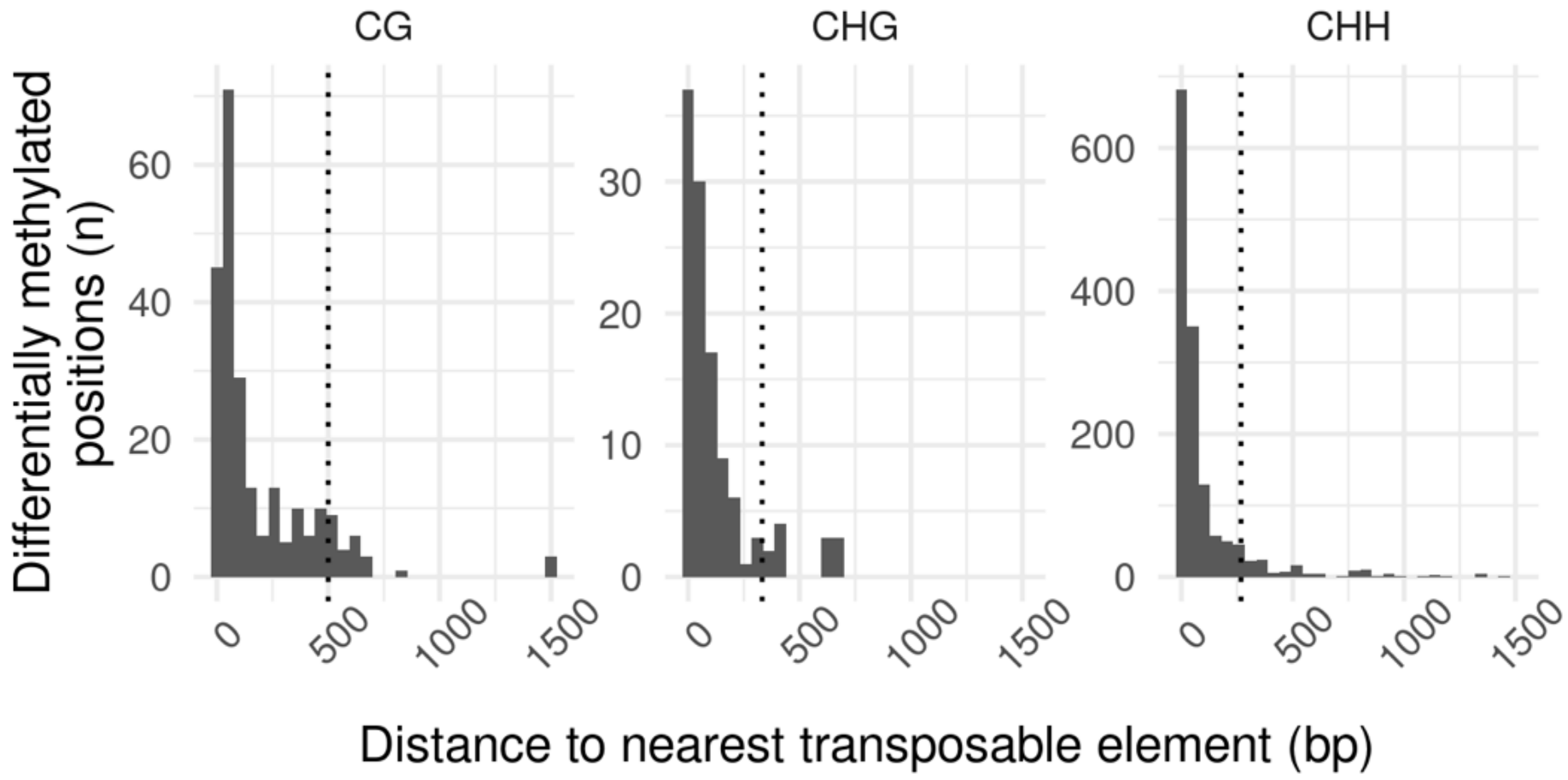

Figure S9. Distance threshold used to define transposable element (TE)-associated CHH differentially methylated positions (DMPs). Histograms show the distance from each DMP to the nearest annotated TE, shown separately for each sequence context. Dotted lines indicate the distance encompassing 90% of DMPs in each context (CG = 500 bp; CHG = 332 bp; CHH = 268 bp). Because 90% of CHH DMPs occurred within 268 bp of a TE, CHH DMPs within this distance were were classified as TE-associated in downstream analyses.

bioRxiv preprint doi: https://doi.org/10.64898/2026.07.14.738593; this version posted September 7, 2026. The copyright holder for this preprint (which was not certified by peer review) is the author/funder, who has granted bioRxiv a license to display the preprint in perpetuity. It is made available under a CC-BY 4.0 International license.

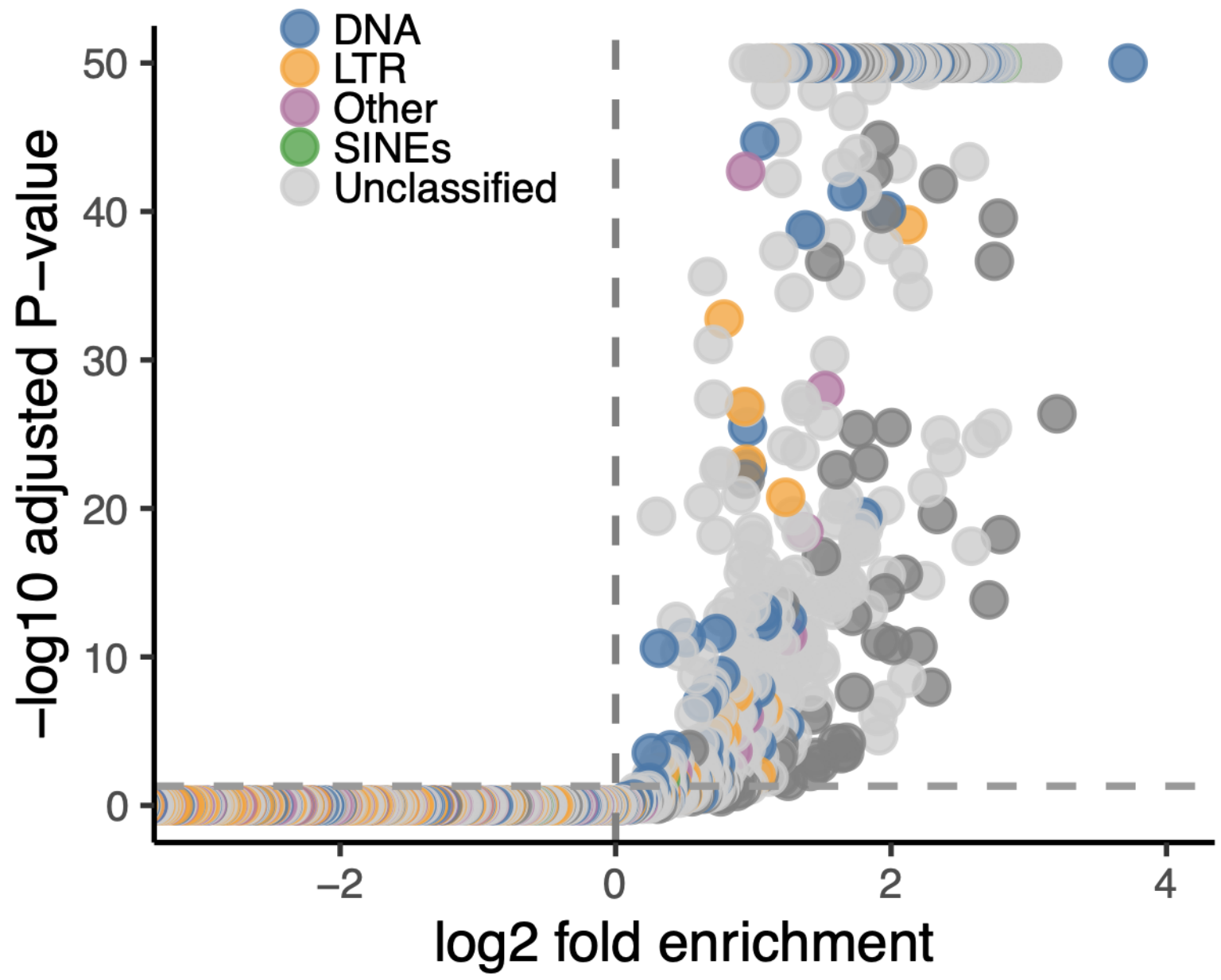

Figure S10. TE family-level enrichment of CHH DMPs. Each point represents a TE family (s1RF level), with log2(fold enrichment) on the x-axis and -log10 adjusted *P*-value (Benjamini-Hochberg) on the y-axis. Points are coloured by TE superfamily.

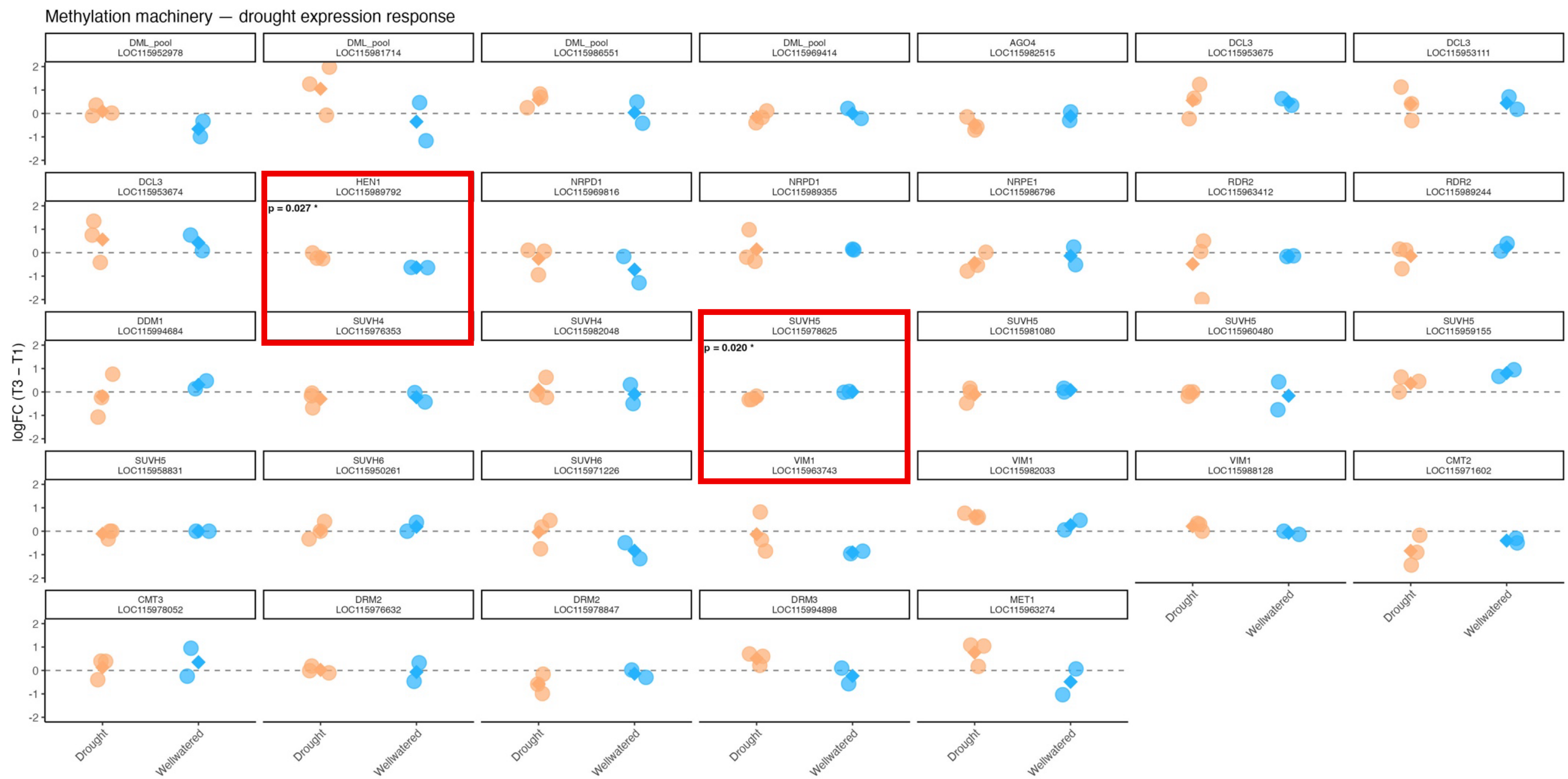

Figure S11. Genes associated with DNA methylation show little transcriptional response to drought. Expression trajectories (T3 – T1 logFC) are shown for 33 genes associated with DNA methylaytion and RNA-directed DNA methylation in five *Q. lobata* seedlings (orange, drought; blue, well-watered). Circles represent individual seedlings and diamonds indicate treatment means. Only HEN1 and an SUVH5 homolog showed significant treatment-specific expression responses (mixed-effects model with *P* values shown), whereas all remaining genes showed no significant transcriptional response (Table S6). These results indicate that despite ~220,000 drought-induced CHH DMPs, expression of the main methylation machinery genes remained stable.

Table S1. The number and direction of differentially methylated positions across all drought-stressed individuals and DNA methylation contexts

Time	Context	Treatment	n_hyper	n_hypo	n_total
T1-T2	CG	Drought	0	4	4
T1-T2	CG	Well-watered	0	9	9
T1-T2	CHG	Drought	2	9	11
T1-T2	CHG	Well-watered	0	5	5
T1-T2	CHH	Drought	8682	66	8748
T1-T2	CHH	Well-watered	7914	80	7994
T1-T3	CG	Drought	3	404	407
T1-T3	CG	Well-watered	1	28	29
T1-T3	CHG	Drought	49	217	266
T1-T3	CHG	Well-watered	0	34	34
T1-T3	CHH	Drought	249756	433	250189
T1-T3	CHH	Well-watered	45448	229	45677

Table S2. Enrichment analysis of genomic features associated with drought-responsive DMPs across sequence contexts. For each feature and sequence context, Fisher's exact tests were used to compare the proportion of DMPs overlapping a given genomic feature relative to the non-DMP genomic background. Fold enrichment values represent the proportion of DMPs within each feature relative to background expectations. P-values were adjusted using the Benjamini-Hochberg procedure. Features were classified as enriched or depleted when adjusted P < 0.05 and fold enrichment values were greater or less than 1, respectively.

Context	feature	DMP	nonDMP	DMP_total	nonDMP_total	a	b	c	d	p_value	odds_ratio	dmp_prop	bg_prop	fold_enrichment	padj	direction
CG	genebody	122	3482275	561	13305859	122	3482275	439	9823584	0.0163298	0.783983	0.217469	0.26171	0.830954	0.0191698	Depleted
CG	upstream	140	1095749	561	13305859	140	1095749	421	12210110	1.24351e-32	3.70553	0.249554	0.0823509	3.03038	3.35747e-32	Enriched
CG	downstream	95	996789	561	13305859	95	996789	466	12309070	1.22336e-13	2.51737	0.16934	0.0749135	2.26048	2.35935e-13	Enriched
CG	introns	62	1697299	561	13305859	62	1697299	499	11608560	0.254316	0.849814	0.110517	0.12756	0.86639	0.264097	NS
CG	exons	55	1696767	561	13305859	55	1696767	506	11609092	0.0365393	0.743684	0.0980392	0.12752	0.768813	0.0411067	Depleted
CG	cds	46	1333137	561	13305859	46	1333137	515	11972722	0.15979	0.802147	0.0819964	0.100192	0.818395	0.172573	NS
CG	TE	202	7455497	561	13305859	202	7455497	359	5850362	1.688e-21	0.44153	0.360071	0.560317	0.642621	3.79801e-21	Depleted
CG	intergenic	221	8023052	561	13305859	221	8023052	340	5282807	2.61374e-23	0.427991	0.393939	0.602971	0.65333	6.41554e-23	Depleted
CG	ACR	0	178668	561	13305859	0	178668	561	13127191	0.00126211	0	0	0.0134278	0	0.00154896	Depleted
CHG	genebody	53	6928015	266	19550156	53	6928015	213	12622141	3.89183e-08	0.453337	0.199248	0.354371	0.562258	7.00529e-08	Depleted
CHG	upstream	64	1360047	266	19550156	64	1360047	202	18190109	1.59091e-18	4.23769	0.240602	0.0695671	3.45855	3.3042e-18	Enriched
CHG	downstream	43	1536771	266	19550156	43	1536771	223	18013385	6.32689e-06	2.2602	0.161654	0.0786066	2.0565	9.49033e-06	Enriched
CHG	introns	28	3762365	266	19550156	28	3762365	238	15787791	0.000171832	0.493682	0.105263	0.192447	0.546973	0.000231973	Depleted
CHG	exons	21	2974380	266	19550156	21	2974380	245	16575776	0.000438568	0.477675	0.0789474	0.152141	0.518909	0.000563874	Depleted
CHG	cds	7	2278830	266	19550156	7	2278830	259	17271326	1.4643e-07	0.204839	0.0263158	0.116563	0.225764	2.47101e-07	Depleted
CHG	TE	129	9297566	266	19550156	129	9297566	137	10252590	0.75936	1.03834	0.484962	0.475575	1.01974	0.75936	NS
CHG	intergenic	102	10231247	266	19550156	102	10231247	164	9318909	5.01022e-06	0.566496	0.383459	0.523333	0.732724	7.95741e-06	Depleted
CHG	ACR	11	177935	266	19550156	11	177935	255	19372221	4.08981e-05	4.69642	0.0413534	0.00910146	4.5436	5.81184e-05	Enriched
CHH	genebody	24018	41526365	250189	135727941	24018	41526365	226171	94201576	0	0.240879	0.0959994	0.305953	0.313772	0	Depleted
CHH	upstream	66814	10580769	250189	135727941	66814	10580769	183375	125147172	0	4.30973	0.267054	0.0779557	3.42572	0	Enriched

Context	feature	DMP	nonDMP	DMP_total	nonDMP_total	a	b	c	d	p_value	odds_ratio	dmp_prop	bg_prop	fold_enrichment	padj	direction
CHH	downstream	41184	11110633	250189	135727941	41184	11110633	209005	124617308	0	2.21006	0.164612	0.0818596	2.0109	0	Enriched
CHH	introns	20839	26030637	250189	135727941	20839	26030637	229350	109697304	0	0.382899	0.083293	0.191785	0.434303	0	Depleted
CHH	exons	1481	14260949	250189	135727941	1481	14260949	248708	121466992	0	0.050693	0.00591952	0.10507	0.0563388	0	Depleted
CHH	cds	583	10264482	250189	135727941	583	10264482	249606	125463459	0	0.0285547	0.00233024	0.0756254	0.0308129	0	Depleted
CHH	TE	226621	65767152	250189	135727941	226621	65767152	23568	69960789	0	10.2287	0.905799	0.484551	1.86936	0	Enriched
CHH	intergenic	124677	75570669	250189	135727941	124677	75570669	125512	60157272	0	0.790749	0.498331	0.55678	0.895023	0	Depleted
CHH	ACR	359	1009295	250189	135727941	359	1009295	249830	134718646	0	0.191807	0.00143492	0.00743616	0.192964	0	Depleted

Table S3. Enrichment analysis of transposable element (TE) families associated with drought-induced CHH DMPs. CHH DMPs located within 268 bp of a TE were assigned to the nearest TE family and tested for enrichment relative to genomic TE abundance using hypergeometric tests. Fold enrichment values represent the observed proportion of TE-associated CHH DMPs relative to the genomic proportion occupied by each TE family. P-values were adjusted using the Benjamini-Hochberg procedure. TE families were considered enriched when adjusted $P < 0.05$ and log2 fold enrichment >1. Only annotated transposable element families are shown.

Family	Superfamily	k	n	expected	Fold enrichment	P-values	Adjusted P-values	log2FE
s1RF1204	DNA/MuLE-MuDR	115	20997	10.7073	10.7403	1.65124e-75	1.72463e-74	3.42496
s1RF1098	Unknown/unclassified	157	31249	15.9353	9.85236	4.69534e-97	5.8548e-96	3.30047
s1RF0893	Unknown/unclassified	302	63644	32.4549	9.30521	4.03191e-178	7.03856e-177	3.21804
s1RF1218	Unknown/unclassified	86	18439	9.40288	9.14614	1.61657e-51	1.30824e-50	3.19316
s1RF0432	Unknown/unclassified	824	188706	96.2297	8.56284	0	0	3.09809
s1RF0447	Unknown/unclassified	814	188239	95.9916	8.47991	0	0	3.08405
s1RF0299	Unknown/unclassified	1209	284150	144.901	8.34363	0	0	3.06068
s1RF0787	Unknown/unclassified	354	83458	42.559	8.31786	4.146e-193	7.79448e-192	3.05621
s1RF0573	Unknown/unclassified	570	134413	68.5433	8.31592	0	3.21481e-308	3.05588
s1RF1266	Tandem/(AACTCAT)n	46	10900	5.5584	8.27576	1.3624e-26	8.12122e-26	3.04889
s1RF0169	Unknown/unclassified	1836	472747	241.075	7.61589	0	0	2.92901
s1RF0649	Unknown/unclassified	453	117069	59.6988	7.5881	1.04727e-230	2.1691e-229	2.92374
s1RF0210	Unknown/unclassified	1511	408408	208.266	7.25516	0	0	2.85901
s1RF0048	SINE/tRNA-Deu-CR1	5630	1536527	783.544	7.1853	0	0	2.84505
s1RF0382	Unknown/unclassified	798	219355	111.859	7.13398	0	0	2.83471
s1RF0374	Unknown/unclassified	812	225858	115.175	7.05013	0	0	2.81765
s1RF0951	Unknown/unclassified	187	52155	26.5962	7.03108	4.68944e-91	5.51009e-90	2.81375
s1RF0356	DNA/MuLE-MuDR	872	244767	124.818	6.98619	0	0	2.80451
s1RF0184	Unknown/unclassified	1661	471369	240.372	6.91011	0	0	2.78871
s1RF0625	Unknown/unclassified	411	116841	59.5825	6.898	8.46648e-195	1.64223e-193	2.78618
s1RF0642	Unknown/unclassified	390	112774	57.5086	6.7816	1.75599e-182	3.15562e-181	2.76163
s1RF0402	Unknown/unclassified	718	207797	105.965	6.77582	0	0	2.7604
s1RF0372	Unknown/unclassified	791	230102	117.339	6.74113	0	0	2.75299
s1RF0046	DNA/unclassified	5695	1658436	845.711	6.73398	0	0	2.75146
s1RF0099	DNA/PIF-Harbinger	2677	780852	398.192	6.72289	0	0	2.74908
s1RF0830	Unknown/unclassified	251	74619	38.0516	6.59631	1.37554e-115	1.97754e-114	2.72166
s1RF1255	Tandem/(AAATCC)n	41	12234	6.23867	6.57191	2.58497e-20	1.36746e-19	2.71631
s1RF0440	Unknown/unclassified	618	186211	94.9574	6.50818	1.42948e-278	3.49365e-277	2.70225
s1RF1045	Unknown/unclassified	127	39113	19.9455	6.36736	8.69638e-58	7.48379e-57	2.67069
s1RF1276	Tandem/(AATAG)n	33	10225	5.21419	6.32888	3.30597e-16	1.55381e-15	2.66195
s1RF1240	Unknown/unclassified	49	15277	7.79043	6.28977	3.6914e-23	2.05977e-22	2.65301
s1RF0271	Unknown/unclassified	1044	326692	166.595	6.26669	0	0	2.6477
s1RF0229	Unknown/unclassified	1185	377301	192.403	6.15895	0	0	2.62269
s1RF1158	Tandem/(AATAAT)n	75	23881	12.178	6.15865	5.90879e-34	3.98925e-33	2.62261
s1RF0135	DNA/MuLE-MuDR	1906	607378	309.729	6.15376	0	0	2.62147
s1RF1127	Tandem/(AAATAAT)n	82	26218	13.3697	6.13325	7.79937e-37	5.38465e-36	2.61665
s1RF1117	Tandem/(AATTAT)n	85	27332	13.9378	6.09851	6.11153e-38	4.34203e-37	2.60846
s1RF0461	Unknown/unclassified	544	175095	89.2888	6.09259	2.62754e-232	5.53595e-231	2.60705
s1RF0870	Unknown/unclassified	212	68679	35.0225	6.05325	2.93912e-91	3.48699e-90	2.59771
s1RF0121	Unknown/unclassified	2051	664710	338.966	6.05076	0	0	2.59712

Family	Superfamily	k	n	expected	Fold enrichment	P-values	Adjusted P-values	log2FE
s1RF0930	Unknown/unclassified	171	55734	28.4213	6.01662	1.31165e-73	1.33569e-72	2.58895
s1RF0194	Unknown/unclassified	1374	449218	229.077	5.998	0	0	2.58448
s1RF1209	Unknown/unclassified	60	19844	10.1194	5.92923	1.10596e-26	6.62494e-26	2.56785
s1RF0016	Tandem/(A)n	11118	3705632	1889.67	5.88357	0	0	2.55669
s1RF0072	Unknown/unclassified	2968	992898	506.323	5.86187	0	0	2.55136
s1RF0131	Unknown/unclassified	1841	633018	322.804	5.70314	0	0	2.51176
s1RF1193	Unknown/unclassified	60	20648	10.5293	5.69836	8.04629e-26	4.68218e-25	2.51055
s1RF1182	Unknown/unclassified	76	26183	13.3519	5.69208	3.26634e-32	2.13448e-31	2.50896
s1RF1275	Tandem/(AATTC)n	29	10011	5.10506	5.68064	2.73123e-13	1.2049e-12	2.50605
s1RF0254	Unknown/unclassified	965	336602	171.649	5.62195	0	0	2.49107
s1RF0822	Unknown/unclassified	210	73336	37.3973	5.61537	1.00271e-84	1.13455e-83	2.48938
s1RF0738	Unknown/unclassified	281	99374	50.6753	5.54511	1.95123e-111	2.70954e-110	2.47122
s1RF0021	Unknown/unclassified	9056	3211313	1637.59	5.53007	0	0	2.4673
s1RF1123	Unknown/unclassified	83	29594	15.0913	5.49985	5.40064e-34	3.66643e-33	2.45939
s1RF0154	Unknown/unclassified	1384	507869	258.985	5.34393	0	0	2.4179
s1RF0214	Unknown/unclassified	1096	402818	205.415	5.33554	0	0	2.41563
s1RF0090	Tandem/(AAT)n	2216	818771	417.528	5.30742	0	0	2.40801
s1RF0119	Tandem/(AAAT)n	1732	644533	328.676	5.26962	0	0	2.3977
s1RF0811	Tandem/(AAATT)n	194	72261	36.8491	5.26471	5.69228e-74	5.84535e-73	2.39635
s1RF0771	Unknown/unclassified	224	85416	43.5575	5.14263	2.93529e-83	3.26084e-82	2.36251
s1RF1236	Tandem/(AAATTC)n	38	14640	7.4656	5.09002	1.96363e-15	9.12377e-15	2.34767
s1RF0660	Unknown/unclassified	288	112017	57.1225	5.04179	1.74254e-104	2.2653e-103	2.33394
s1RF0888	Unknown/unclassified	162	63211	32.2341	5.02573	1.91234e-59	1.70575e-58	2.32933
s1RF0443	Unknown/unclassified	472	184612	94.142	5.0137	4.94343e-169	8.39011e-168	2.32588
s1RF0350	DNA/hAT-Ac	609	238202	121.47	5.01359	1.66372e-217	3.3329e-216	2.32584
s1RF0141	Unknown/unclassified	1492	587088	299.383	4.98359	0	0	2.31718
s1RF0219	Unknown/unclassified	990	393548	200.688	4.93303	0	0	2.30247
s1RF0859	Tandem/(AAAATT)n	164	65447	33.3744	4.91395	7.16733e-59	6.21169e-58	2.29688
s1RF0401	DNA/hAT-Ac	516	205999	105.048	4.91203	6.31684e-181	1.11872e-179	2.29632
s1RF0202	Unknown/unclassified	1036	413601	210.914	4.91196	0	0	2.2963
s1RF0285	Unknown/unclassified	732	292918	149.372	4.90051	3.11378e-255	6.79471e-254	2.29293
s1RF0823	Unknown/unclassified	188	75713	38.6095	4.86927	1.30538e-66	1.23657e-65	2.28371
s1RF0745	Tandem/(AAAAAAT)n	218	88788	45.277	4.81481	4.40181e-76	4.63708e-75	2.26748
s1RF0854	Unknown/unclassified	169	69188	35.2821	4.78997	4.10099e-59	3.60533e-58	2.26002
s1RF0949	Unknown/unclassified	133	54494	27.7889	4.78608	6.93807e-47	5.33227e-46	2.25884
s1RF1229	Tandem/(AGAT)n	38	15600	7.95514	4.77678	1.36955e-14	6.22151e-14	2.25604
s1RF0260	DNA/hAT-Tip100	794	329010	167.777	4.73247	3.94619e-267	9.27355e-266	2.24259
s1RF1092	Tandem/(AATATAT)n	72	29894	15.2443	4.72308	7.10632e-26	4.15499e-25	2.23973
s1RF1100	Unknown/unclassified	77	32000	16.3182	4.71864	1.58735e-27	9.69868e-27	2.23837
s1RF0314	Unknown/unclassified	653	272793	139.109	4.69415	3.22709e-218	6.5725e-217	2.23086
s1RF0671	Unknown/unclassified	251	105136	53.6136	4.68165	6.98358e-85	7.97564e-84	2.22702
s1RF0522	Unknown/unclassified	360	150951	76.9767	4.67674	9.49415e-121	1.41486e-119	2.2255
s1RF0093	Unknown/unclassified	1933	812279	414.218	4.66663	0	0	2.22238
s1RF0697	Unknown/unclassified	242	101940	51.9838	4.6553	1.92629e-81	2.10172e-80	2.21887
s1RF0571	Unknown/unclassified	323	136162	69.4351	4.65182	5.91206e-108	8.02726e-107	2.2178
s1RF0566	Tandem/(AAAAG)n	313	132063	67.3449	4.64772	1.40126e-104	1.84122e-103	2.21652
s1RF0031	Tandem/(AT)n	5018	2120320	1081.25	4.64094	0	0	2.21442
s1RF0712	DNA/hAT-Tip100	250	106112	54.1113	4.62011	2.02118e-83	2.26595e-82	2.20793

Family	Superfamily	k	n	expected	Fold enrichment	P-values	Adjusted P-values	log2FE
s1RF1130	Unknown/unclassified	67	28805	14.689	4.56125	2.13611e-23	1.1974e-22	2.18943
s1RF0125	Unknown/unclassified	1438	630814	321.681	4.47027	0	0	2.16036
s1RF0704	Unknown/unclassified	227	100325	51.1602	4.43704	8.22713e-73	8.24061e-72	2.1496
s1RF0947	Tandem/(ATAT)n	115	51038	26.0266	4.41856	1.18393e-37	8.31471e-37	2.14358
s1RF0905	Unknown/unclassified	135	60454	30.8282	4.3791	1.78997e-43	1.33375e-42	2.13064
s1RF0149	Unknown/unclassified	1217	546675	278.774	4.36554	0	0	2.12616
s1RF0079	Unknown/unclassified	2091	942863	480.808	4.34893	0	0	2.12066
s1RF1252	Unknown/unclassified	29	13117	6.68895	4.33551	1.52728e-10	6.22112e-10	2.1162
s1RF0667	Unknown/unclassified	238	107682	54.9119	4.33421	2.28261e-74	2.36385e-73	2.11577
s1RF0179	DNA/TcMar-Stowaway	999	452026	230.508	4.3339	8.36101e-306	2.12857e-304	2.11567
s1RF0881	LTR/Copia	144	65188	33.2423	4.33183	1.00865e-45	7.70356e-45	2.11498
s1RF0221	DNA/PIF-Harbinger	867	393370	200.597	4.3221	7.56699e-265	1.74469e-263	2.11173
s1RF0532	Unknown/unclassified	325	150367	76.6789	4.23845	2.09115e-98	2.63442e-97	2.08354
s1RF0116	Unknown/unclassified	1469	682136	347.852	4.22306	0	0	2.07829
s1RF0034	DNA/hAT-Tip100	4337	2014184	1027.12	4.22247	0	0	2.07809
s1RF0239	Unknown/unclassified	782	363333	185.28	4.22064	7.11918e-233	1.52625e-231	2.07746
s1RF1118	Tandem/(AATATT)n	57	26753	13.6426	4.1781	1.80122e-18	9.05801e-18	2.06285
s1RF0049	Unknown/unclassified	3190	1499609	764.718	4.17147	0	0	2.06056
s1RF0145	Unknown/unclassified	1221	578311	294.907	4.14029	0	0	2.04973
s1RF0682	Unknown/unclassified	223	106068	54.0889	4.12285	4.28043e-66	4.02361e-65	2.04364
s1RF0964	Tandem/(AAA)n	105	50038	25.5166	4.11496	4.86667e-32	3.16334e-31	2.04088
s1RF0148	Unknown/unclassified	1152	550178	280.561	4.10606	0	0	2.03776
s1RF0146	Unknown/unclassified	1252	601515	306.74	4.08164	0	0	2.02915
s1RF0990	Unknown/unclassified	99	47952	24.4529	4.0486	8.84045e-30	5.54001e-29	2.01742
s1RF0297	Tandem/(AAAAT)n	581	281803	143.704	4.04303	2.78764e-165	4.66643e-164	2.01544
s1RF1188	Tandem/(AAATATT)n	42	20444	10.4253	4.02865	1.55885e-13	6.92694e-13	2.0103
s1RF0788	Unknown/unclassified	164	80586	41.0944	3.99081	2.32757e-47	1.80019e-46	1.99668
s1RF1248	Tandem/(AATAC)n	27	13285	6.77462	3.98546	3.68786e-09	1.4398e-08	1.99475
s1RF0691	DNA/hAT-Tip100	213	105119	53.6049	3.97352	1.22227e-60	1.12302e-59	1.99042
s1RF0422	Unknown/unclassified	399	198245	101.094	3.94682	5.34751e-111	7.34231e-110	1.98069
s1RF0429	Unknown/unclassified	384	193611	98.731	3.88936	4.49222e-105	5.96684e-104	1.95953
s1RF0340	DNA/PIF-Harbinger	489	248020	126.477	3.86633	2.56092e-132	3.96132e-131	1.95096
s1RF0380	Unknown/unclassified	431	221561	112.984	3.8147	5.378e-115	7.64176e-114	1.93157
s1RF0664	Unknown/unclassified	226	117328	59.8308	3.77732	1.60008e-60	1.45917e-59	1.91736
s1RF0539	Tandem/(AAAAAG)n	267	139053	70.9094	3.76537	7.04211e-71	6.82973e-70	1.91279
s1RF0982	Unknown/unclassified	95	49580	25.2831	3.75745	2.406e-26	1.42725e-25	1.90976
s1RF0734	Tandem/(AATAT)n	169	88529	45.1449	3.7435	2.98707e-45	2.25321e-44	1.90439
s1RF0798	Unknown/unclassified	156	81740	41.6829	3.74254	6.68511e-42	4.89174e-41	1.90402
s1RF0478	Unknown/unclassified	332	174779	89.1277	3.72499	1.64056e-86	1.9093e-85	1.89724
s1RF0777	DNA/MuLE-MuDR	159	83932	42.8007	3.71489	2.7011e-42	1.9884e-41	1.89332
s1RF0611	Unknown/unclassified	228	120742	61.5718	3.70299	1.35645e-59	1.21881e-58	1.88869
s1RF0668	Tandem/(AATT)n	195	103550	52.8048	3.69284	4.47706e-51	3.59932e-50	1.88473
s1RF0683	Tandem/(AA)n	190	101747	51.8854	3.66192	2.62574e-49	2.0701e-48	1.8726
s1RF0911	Tandem/(AAATAT)n	109	58721	29.9445	3.64007	7.3007e-29	4.52865e-28	1.86397
s1RF0580	Unknown/unclassified	237	129093	65.8303	3.60016	9.30326e-60	8.42117e-59	1.84806
s1RF0970	Unknown/unclassified	95	52049	26.5421	3.57921	7.06553e-25	4.07268e-24	1.83964
s1RF0238	Unknown/unclassified	796	438437	223.579	3.56027	2.9022e-193	5.5414e-192	1.83198

Family	Superfamily	k	n	expected	Fold enrichment	P-values	Adjusted P-values	log2FE
s1RF0650	Unknown/unclassified	207	114089	58.1791	3.55798	1.13917e-51	9.2804e-51	1.83106
s1RF0191	LTR/Copia	774	427140	217.818	3.55343	1.64709e-187	3.0041e-186	1.82921
s1RF0363	Unknown/unclassified	422	234365	119.513	3.53099	2.6511e-102	3.41015e-101	1.82007
s1RF0770	Tandem/(AAAC)n	151	84212	42.9435	3.51625	1.21652e-37	8.49478e-37	1.81404
s1RF0871	Unknown/unclassified	120	67019	34.176	3.51123	3.1059e-30	1.96653e-29	1.81198
s1RF0132	Unknown/unclassified	1110	620428	316.384	3.50839	1.10319e-263	2.45109e-262	1.81081
s1RF0170	Unknown/unclassified	820	462659	235.931	3.4756	7.00413e-193	1.29683e-191	1.79726
s1RF1017	Unknown/unclassified	75	42551	21.6987	3.45643	3.48803e-19	1.77599e-18	1.78928
s1RF0460	Tandem/(AAAAAT)n	304	173167	88.3057	3.44259	7.42032e-72	7.37206e-71	1.78349
s1RF0259	Unknown/unclassified	573	327264	166.887	3.43347	3.2703e-133	5.12347e-132	1.77967
s1RF1247	Unknown/unclassified	25	14296	7.29018	3.42927	2.22574e-07	8.24199e-07	1.7779
s1RF0607	DNA/hAT-Tag1	211	121367	61.8905	3.40925	7.86307e-50	6.2394e-49	1.76945
s1RF0458	Unknown/unclassified	310	179259	91.4123	3.39123	8.45485e-72	8.33212e-71	1.76181
s1RF0511	Unknown/unclassified	264	152866	77.9533	3.38664	2.52078e-61	2.35144e-60	1.75986
s1RF1251	Unknown/unclassified	24	13919	7.09793	3.38127	4.89677e-07	1.80237e-06	1.75756
s1RF0110	Unknown/unclassified	1203	702300	358.134	3.35907	1.75444e-269	4.20377e-268	1.74806
s1RF0543	Unknown/unclassified	249	146021	74.4627	3.34396	5.54349e-57	4.73716e-56	1.74156
s1RF0972	Unknown/unclassified	86	50521	25.7629	3.33813	8.04715e-21	4.33199e-20	1.73904
s1RF1150	Tandem/(AAAATAT)n	41	24139	12.3096	3.33074	9.34455e-11	3.83189e-10	1.73584
s1RF1081	Unknown/unclassified	56	33140	16.8996	3.31369	5.12904e-14	2.29586e-13	1.72844
s1RF0963	Unknown/unclassified	99	58623	29.8945	3.31164	1.82937e-23	1.03018e-22	1.72755
s1RF1297	Unknown/unclassified	17	10096	5.14841	3.30199	2.82771e-05	9.81665e-05	1.72334
s1RF0815	Tandem/(AAAAAC)n	123	73176	37.3157	3.2962	1.51027e-28	9.32093e-28	1.7208
s1RF0525	Tandem/(AAAG)n	244	146607	74.7615	3.26371	4.47865e-54	3.69791e-53	1.70651
s1RF1000	Unknown/unclassified	76	46003	23.459	3.23969	6.62914e-18	3.27968e-17	1.69586
s1RF0622	Unknown/unclassified	196	119328	60.8507	3.221	4.9432e-43	3.66096e-42	1.68751
s1RF1159	Unknown/unclassified	43	26292	13.4075	3.20717	1.04762e-10	4.28157e-10	1.6813
s1RF1037	Tandem/(AAAAATT)n	63	38620	19.6941	3.19893	6.90694e-15	3.14936e-14	1.67759
s1RF0451	Tandem/(C)n	286	175796	89.6463	3.19032	4.29097e-61	3.9724e-60	1.6737
s1RF0482	Unknown/unclassified	278	171384	87.3964	3.18091	3.41015e-59	3.01971e-58	1.66944
s1RF0835	Unknown/unclassified	119	74270	37.8736	3.14203	5.84322e-26	3.44948e-25	1.6517
s1RF0027	Unknown/unclassified	4042	2531887	1291.12	3.13061	0	0	1.64644
s1RF0517	Unknown/unclassified	242	152382	77.7065	3.11428	2.97101e-50	2.37293e-49	1.6389
s1RF0928	Unknown/unclassified	91	57529	29.3366	3.10192	6.4692e-20	3.36398e-19	1.63316
s1RF0974	Unknown/unclassified	79	49955	25.4743	3.10116	1.65054e-17	8.06786e-17	1.63281
s1RF0258	DNA/MuLE-MuDR	527	333784	170.212	3.09615	3.1812e-106	4.2719e-105	1.63047
s1RF0967	Unknown/unclassified	81	51416	26.2193	3.08932	8.11473e-18	3.99847e-17	1.62729
s1RF0330	Unknown/unclassified	411	261260	133.228	3.08493	7.69957e-83	8.47647e-82	1.62524
s1RF0853	Unknown/unclassified	111	70836	36.1225	3.07288	1.33523e-23	7.58908e-23	1.61959
s1RF0833	Unknown/unclassified	112	72092	36.763	3.04654	1.63206e-23	9.2332e-23	1.60717
s1RF0203	Unknown/unclassified	726	468750	239.037	3.03719	1.72279e-141	2.73408e-140	1.60274
s1RF0932	Unknown/unclassified	89	57553	29.3489	3.03248	6.3943e-19	3.24225e-18	1.6005
s1RF0987	Unknown/unclassified	75	48572	24.7691	3.02797	3.53326e-16	1.65427e-15	1.59835
s1RF0636	LINE/L1	188	122182	62.3061	3.01736	1.03099e-37	7.28246e-37	1.59329
s1RF0186	Unknown/unclassified	674	446251	227.564	2.96181	1.40716e-126	2.14944e-125	1.56648
s1RF0634	Unknown/unclassified	173	115279	58.786	2.94288	1.34614e-33	9.03837e-33	1.55723
s1RF0953	DNA/unclassified	79	52728	26.8884	2.93807	2.94866e-16	1.39347e-15	1.55487
s1RF0419	DNA/hAT-Ac	292	194898	99.3873	2.938	2.84013e-55	2.36098e-54	1.55484

Family	Superfamily	k	n	expected	Fold enrichment	P-values	Adjusted P-values	log2FE
s1RF0560	DNA/hAT-Ac	205	137205	69.967	2.92995	3.29221e-39	2.39469e-38	1.55088
s1RF1176	Tandem/(AAATTAT)n	33	22154	11.2973	2.92105	1.17697e-07	4.37159e-07	1.54648
s1RF0992	Unknown/unclassified	73	49218	25.0985	2.90854	6.16921e-15	2.82351e-14	1.5403
s1RF0045	Unknown/unclassified	2530	1711607	872.826	2.89863	0	0	1.53537
s1RF0910	Unknown/unclassified	92	62384	31.8124	2.89195	3.11249e-18	1.5588e-17	1.53204
s1RF0469	Unknown/unclassified	261	177246	90.3857	2.88762	2.57278e-48	2.01535e-47	1.52988
s1RF0251	DNA/hAT-Tip100	520	356621	181.857	2.85939	7.09242e-93	8.58113e-92	1.51571
s1RF1015	Unknown/unclassified	65	44657	22.7726	2.8543	3.95753e-13	1.72718e-12	1.51314
s1RF0282	Tandem/(AAG)n	429	295848	150.866	2.84358	2.69886e-76	2.89299e-75	1.50771
s1RF0975	Unknown/unclassified	77	53211	27.1347	2.8377	4.04573e-15	1.87268e-14	1.50472
s1RF1090	Unknown/unclassified	47	32585	16.6166	2.8285	8.15866e-10	3.24752e-09	1.50004
s1RF1134	Unknown/unclassified	39	27075	13.8068	2.8247	2.1546e-08	8.12628e-08	1.4981
s1RF0060	DNA/hAT-Tip100	1785	1242177	633.442	2.81794	5.66665e-307	1.47333e-305	1.49464
s1RF0028	Composition/A-RICH	3338	2325696	1185.98	2.81456	0	0	1.49291
s1RF0802	Tandem/(AAAAC)n	109	76091	38.8022	2.80912	2.20689e-20	1.17253e-19	1.49012
s1RF1238	Tandem/(AAAAA)n	21	14678	7.48497	2.80562	3.73498e-05	0.000129296	1.48832
s1RF0378	DNA/hAT-Tag1	317	222593	113.51	2.7927	2.81691e-55	2.35772e-54	1.48166
s1RF0085	DNA/MuLE-MuDR	1238	869662	443.48	2.79156	4.28049e-210	8.43671e-209	1.48107
s1RF1263	Tandem/(AAACCC)n	16	11283	5.75371	2.78081	0.000324792	0.00106122	1.47551
s1RF0163	DNA/hAT-Ac	680	481741	245.661	2.76804	1.20795e-114	1.69668e-113	1.46886
s1RF0381	Unknown/unclassified	329	234652	119.66	2.74946	7.26402e-56	6.12181e-55	1.45915
s1RF0152	Unknown/unclassified	730	523572	266.993	2.73415	1.94919e-120	2.86977e-119	1.45109
s1RF1264	Tandem/(AATGAT)n	16	11492	5.86029	2.73024	0.000395155	0.00128085	1.44903
s1RF0594	Unknown/unclassified	173	124629	63.554	2.7221	8.71955e-30	5.49242e-29	1.44472
s1RF0199	DNA/hAT-Ac	565	410049	209.102	2.70202	8.06366e-92	9.66059e-91	1.43404
s1RF0275	Unknown/unclassified	437	317441	161.877	2.69957	2.34847e-71	2.29587e-70	1.43273
s1RF0196	Unknown/unclassified	593	431311	219.945	2.69613	6.44533e-96	7.95575e-95	1.43089
s1RF0109	Unknown/unclassified	950	700836	357.388	2.65818	8.17849e-149	1.31502e-147	1.41044
s1RF0857	Unknown/unclassified	94	69655	35.5202	2.64638	2.95342e-16	1.39347e-15	1.40402
s1RF1102	Tandem/(AATAATT)n	40	29795	15.1938	2.63265	8.89646e-08	3.31448e-07	1.39652
s1RF0050	Tandem/(AG)n	1963	1464306	746.716	2.62885	7.34197e-300	1.831e-298	1.39443
s1RF0981	Unknown/unclassified	68	50826	25.9185	2.62361	4.65701e-12	1.9968e-11	1.39155
s1RF1254	Tandem/(AATG)n	16	11988	6.11322	2.61728	0.000616483	0.00198248	1.38807
s1RF0542	Unknown/unclassified	195	146459	74.6861	2.61093	4.03456e-31	2.59486e-30	1.38456
s1RF0672	LINE/L1	141	106413	54.2648	2.59837	8.08988e-23	4.45308e-22	1.37761
s1RF0730	Unknown/unclassified	125	94711	48.2974	2.58813	2.69457e-20	1.41929e-19	1.37191
s1RF0488	Tandem/(AAC)n	213	161428	82.3194	2.58748	2.62898e-33	1.73655e-32	1.37155
s1RF0503	Unknown/unclassified	210	159299	81.2337	2.58513	8.20313e-33	5.38937e-32	1.37024
s1RF0698	Unknown/unclassified	145	110659	56.43	2.56955	5.55774e-23	3.07311e-22	1.36152
s1RF0092	Unknown/unclassified	1191	912754	465.454	2.55879	6.61907e-174	1.13923e-172	1.35546
s1RF0051	Unknown/unclassified	1814	1390993	709.33	2.55734	8.79233e-264	1.98967e-262	1.35465
s1RF0094	LTR/Copia	1055	809700	412.903	2.55508	7.56214e-154	1.23212e-152	1.35337
s1RF0373	Unknown/unclassified	302	232780	118.705	2.54412	4.43564e-45	3.32537e-44	1.34717
s1RF0776	Composition/G-RICH	107	82680	42.1623	2.53781	4.6351e-17	2.23877e-16	1.34359
s1RF0907	Unknown/unclassified	81	62633	31.9394	2.53605	2.6184e-13	1.15931e-12	1.34259
s1RF0794	Unknown/unclassified	102	79003	40.2872	2.53182	2.8034e-16	1.33298e-15	1.34018
s1RF1041	Unknown/unclassified	51	39510	20.1479	2.53128	6.00847e-09	2.32353e-08	1.33987
s1RF1137	Tandem/(AAAAAAC)n	33	25665	13.0877	2.52144	2.73474e-06	9.743e-06	1.33425

Family	Superfamily	k	n	expected	Fold enrichment	P-values	Adjusted P-values	log2FE
s1RF0957	Unknown/unclassified	67	52221	26.6298	2.51597	3.78233e-11	1.58288e-10	1.33112
s1RF0657	Unknown/unclassified	142	112101	57.1654	2.48402	2.85656e-21	1.55143e-20	1.31268
s1RF0709	Unknown/unclassified	128	101135	51.5733	2.4819	2.56385e-19	1.3164e-18	1.31145
s1RF1140	Unknown/unclassified	34	26904	13.7196	2.47821	2.81167e-06	9.98795e-06	1.3093
s1RF0108	Unknown/unclassified	906	718259	366.273	2.47357	1.12714e-124	1.70045e-123	1.30659
s1RF0412	Unknown/unclassified	261	207060	105.589	2.47184	3.05411e-37	2.12053e-36	1.30559
s1RF0646	Tandem/(ATC)n	137	109218	55.6952	2.45982	3.11176e-20	1.62503e-19	1.29855
s1RF0201	Unknown/unclassified	541	433931	221.281	2.44486	1.34316e-73	1.35649e-72	1.28975
s1RF0644	Unknown/unclassified	143	114988	58.6376	2.43871	1.00771e-20	5.40098e-20	1.28612
s1RF0583	Unknown/unclassified	161	129494	66.0348	2.43811	4.24444e-23	2.35759e-22	1.28576
s1RF1042	Unknown/unclassified	52	42089	21.4631	2.42276	1.72362e-08	6.52095e-08	1.27665
s1RF0666	Unknown/unclassified	136	110590	56.3948	2.41157	2.10927e-19	1.08756e-18	1.26997
s1RF0923	Unknown/unclassified	73	59419	30.3004	2.40921	3.58126e-11	1.50388e-10	1.26856
s1RF0253	Unknown/unclassified	447	365114	186.188	2.4008	4.55591e-59	3.97666e-58	1.26351
s1RF0681	Unknown/unclassified	128	104699	53.3907	2.39742	3.59922e-18	1.7952e-17	1.26148
s1RF0479	Unknown/unclassified	202	165882	84.5907	2.38797	1.90297e-27	1.15693e-26	1.25578
s1RF1009	DNA/MuLE-MuDR	56	46051	23.4835	2.38465	8.5782e-09	3.3068e-08	1.25378
s1RF1129	Tandem/(AACAAT)n	32	26534	13.5309	2.36496	1.33795e-05	4.67135e-05	1.24182
s1RF0071	LTR/Gypsy	1227	1018019	519.134	2.36355	6.38253e-154	1.05398e-152	1.24096
s1RF1179	Tandem/(ACATAT)n	26	21671	11.051	2.35272	8.77191e-05	0.000297758	1.23433
s1RF1269	Tandem/(AAAAGT)n	13	10894	5.55534	2.34009	0.00481273	0.0147029	1.22656
s1RF0935	Unknown/unclassified	70	58713	29.9404	2.33798	3.04928e-10	1.22573e-09	1.22526
s1RF0504	LTR/Copia	187	157479	80.3056	2.3286	2.34306e-24	1.34423e-23	1.21946
s1RF0147	Unknown/unclassified	648	546131	278.497	2.32678	1.20245e-79	1.30034e-78	1.21833
s1RF0917	Unknown/unclassified	72	60917	31.0643	2.31777	2.48367e-10	1.00166e-09	1.21274
s1RF0592	Unknown/unclassified	155	131365	66.9889	2.31381	3.01926e-20	1.58349e-19	1.21027
s1RF1227	Unknown/unclassified	20	16956	8.64663	2.31304	0.000654663	0.00208877	1.20979
s1RF0081	DNA/hAT-Ac	1026	880811	449.165	2.28424	2.61726e-120	3.80749e-119	1.19171
s1RF1277	Tandem/(ACCATC)n	12	10308	5.25651	2.28288	0.0078995	0.0236598	1.19086
s1RF1187	Tandem/(ACTCATC)n	24	20651	10.5309	2.27901	0.000251589	0.00082424	1.18841
s1RF0914	Unknown/unclassified	69	59564	30.3744	2.27165	1.27439e-09	5.0562e-09	1.18374
s1RF0779	LINE/L2	101	87598	44.6702	2.26102	3.27042e-13	1.43242e-12	1.17697
s1RF0614	Unknown/unclassified	160	138904	70.8334	2.25882	6.8061e-20	3.52418e-19	1.17557
s1RF1044	DNA/CMC-EnSpm	45	39080	19.9287	2.25805	9.58143e-07	3.48467e-06	1.17508
s1RF0107	Unknown/unclassified	831	726976	370.718	2.2416	5.39624e-94	6.59421e-93	1.16453
s1RF0448	Unknown/unclassified	206	183970	93.8146	2.19582	1.07467e-23	6.13664e-23	1.13476
s1RF0756	DNA/CMC-EnSpm	102	91246	46.5305	2.19211	1.46785e-12	6.3382e-12	1.13232
s1RF0177	Unknown/unclassified	517	463783	236.504	2.18601	4.16998e-56	3.5387e-55	1.1283
s1RF0457	Unknown/unclassified	201	181079	92.3404	2.17673	9.55077e-23	5.23365e-22	1.12216
s1RF0089	Unknown/unclassified	931	838999	427.843	2.17603	1.76587e-98	2.24781e-97	1.1217
s1RF0718	Unknown/unclassified	106	95554	48.7273	2.17537	8.58035e-13	3.71815e-12	1.12126
s1RF1141	Unknown/unclassified	29	26183	13.3519	2.17198	0.000138415	0.00046468	1.11901
s1RF0633	Unknown/unclassified	129	117211	59.7712	2.15823	5.81379e-15	2.67085e-14	1.10985
s1RF1161	Unknown/unclassified	28	25609	13.0592	2.14408	0.000218442	0.000725371	1.10036
s1RF0605	Unknown/unclassified	138	126884	64.7039	2.13279	1.67407e-15	7.80805e-15	1.09274
s1RF0464	Unknown/unclassified	195	179898	91.7381	2.12562	4.92599e-21	2.66352e-20	1.08788
s1RF0175	Unknown/unclassified	510	471091	240.231	2.12296	6.9783e-52	5.72314e-51	1.08608
s1RF0248	Unknown/unclassified	371	343170	174.998	2.12003	3.83125e-38	2.73789e-37	1.08408

Family	Superfamily	k	n	expected	Fold enrichment	P-values	Adjusted P-values	log2FE
s1RF0920	Unknown/unclassified	64	59499	30.3412	2.10934	6.80752e-08	2.54397e-07	1.07679
s1RF0541	DNA/hAT-Tag1	152	141757	72.2883	2.10269	2.12074e-16	1.01629e-15	1.07224
s1RF0118	Composition/GA-RICH	691	645086	328.958	2.10057	6.29827e-68	6.01288e-67	1.07078
s1RF0973	Unknown/unclassified	54	50428	25.7155	2.0999	7.64247e-07	2.78779e-06	1.07032
s1RF0832	Unknown/unclassified	79	73872	37.6707	2.09712	2.87358e-09	1.1291e-08	1.06841
s1RF0096	Unknown/unclassified	882	828237	422.355	2.08829	5.43256e-85	6.26282e-84	1.06232
s1RF0249	LTR/Gypsy	362	340046	173.405	2.0876	5.52192e-36	3.79089e-35	1.06185
s1RF0760	Unknown/unclassified	97	91123	46.4677	2.08747	6.5664e-11	2.70173e-10	1.06176
s1RF0895	DNA/PIF-Harbinger	73	68854	35.1118	2.07908	1.54212e-08	5.88895e-08	1.05594
s1RF0263	Unknown/unclassified	363	342556	174.685	2.07803	1.06389e-35	7.26301e-35	1.05522
s1RF0847	Unknown/unclassified	73	68899	35.1347	2.07772	1.58168e-08	6.02124e-08	1.055
s1RF0897	LINE/L1	68	64276	32.7772	2.07461	5.00463e-08	1.88174e-07	1.05284
s1RF0686	Unknown/unclassified	110	104057	53.0634	2.07299	5.6371e-12	2.40858e-11	1.05172
s1RF0102	Unknown/unclassified	809	768067	391.672	2.0655	4.15186e-76	4.4118e-75	1.04649
s1RF0701	Unknown/unclassified	108	102599	52.3199	2.06423	1.10457e-11	4.68673e-11	1.0456
s1RF0252	Tandem/(AC)n	348	331494	169.044	2.05864	1.44987e-33	9.62903e-33	1.04169
s1RF0354	Unknown/unclassified	255	243094	123.965	2.05704	4.83394e-25	2.79956e-24	1.04057
s1RF0801	Unknown/unclassified	82	78327	39.9425	2.05295	3.70048e-09	1.44012e-08	1.0377
s1RF1173	Tandem/(ACTC)n	24	22971	11.7139	2.04884	0.00107374	0.00339925	1.03481
s1RF1002	Unknown/unclassified	50	47871	24.4116	2.04821	3.69718e-06	1.30201e-05	1.03436
s1RF0267	Unknown/unclassified	332	318182	162.255	2.04616	1.17927e-31	7.62472e-31	1.03292
s1RF1080	Unknown/unclassified	36	34574	17.6308	2.04188	8.10345e-05	0.000276604	1.0299
s1RF0719	DNA/hAT-Tip100	104	100239	51.1164	2.03457	5.69679e-11	2.35982e-10	1.02473
s1RF0956	Unknown/unclassified	55	53014	27.0342	2.03446	1.53867e-06	5.56289e-06	1.02464
s1RF0231	LTR/Copia	425	411868	210.03	2.02352	6.03015e-39	4.36027e-38	1.01687
s1RF0556	LTR/Copia	141	136830	69.7758	2.02076	4.56237e-14	2.04971e-13	1.0149
s1RF0767	Unknown/unclassified	89	86401	44.0598	2.01998	1.77107e-09	6.98143e-09	1.01434
s1RF0508	Unknown/unclassified	167	162333	82.7809	2.01737	2.77103e-16	1.32273e-15	1.01248
s1RF1242	Tandem/(AATC)n	14	13628	6.94953	2.01452	0.0120886	0.0355102	1.01044
s1RF0725	DNA/hAT-Ac	96	93625	47.7436	2.01074	5.3414e-10	2.13307e-09	1.00773
s1RF0673	Unknown/unclassified	108	105371	53.7334	2.00992	4.90862e-11	2.04025e-10	1.00714
s1RF1194	Unknown/unclassified	23	22441	11.4437	2.00984	0.00170801	0.00531092	1.00708
s1RF0150	DNA/hAT-Ac	536	524255	267.341	2.00493	1.56087e-47	1.2149e-46	1.00355

Table S4. Gene Ontology (GO) biological process gene set enrichment analysis (GSEA) of drought-responsive transcriptional responses. Genes were ranked according to the mean interaction effect between drought and well-watered treatments across genomic feature classes, calculated from expression models. Valley oak genes were mapped to their best Arabidopsis thaliana TAIR orthologues prior to analysis. The table reports the top enriched GO biological process terms ranked by nominal P value, including GO identifier (ID), term description, nominal P value, Benjamini–Hochberg adjusted P value (Padj), and normalised enrichment score (NES). Positive NES values indicate enrichment among genes upregulated under drought, whereas negative NES values indicate enrichment among genes relatively downregulated under drought. Only the top 30 enriched GO terms are shown.

ID	Description	pvalue	p.adjust	NES
GO:0019752	carboxylic acid metabolic process	2.0367e-05	0.0110369	1.69231
GO:0006082	organic acid metabolic process	2.95483e-05	0.0110369	1.65564
GO:0043436	oxoacid metabolic process	2.95483e-05	0.0110369	1.65564
GO:0070592	cell wall polysaccharide biosynthetic process	3.57853e-05	0.0110369	2.08159
GO:0015979	photosynthesis	4.28451e-05	0.0110369	-1.97245
GO:0045489	pectin biosynthetic process	5.60882e-05	0.0120403	1.9967
GO:0032787	monocarboxylic acid metabolic process	9.04303e-05	0.0166392	1.75118
GO:0044036	cell wall macromolecule metabolic process	0.00012635	0.0170252	1.88997
GO:0010025	wax biosynthetic process	0.000132184	0.0170252	2.01616
GO:0010166	wax metabolic process	0.000132184	0.0170252	2.01616
GO:0000271	polysaccharide biosynthetic process	0.000157346	0.0178401	1.83401
GO:0010383	cell wall polysaccharide metabolic process	0.000166213	0.0178401	1.9145
GO:0045491	xylan metabolic process	0.000188514	0.0186774	1.91858
GO:0016192	vesicle-mediated transport	0.000253767	0.0233465	1.66351
GO:0031647	regulation of protein stability	0.000309672	0.0265905	1.98721
GO:0019684	photosynthesis, light reaction	0.000383369	0.0308612	-1.91273
GO:0046907	intracellular transport	0.000825894	0.0585092	1.55939
GO:0052325	cell wall pectin biosynthetic process	0.000843333	0.0585092	1.8875
GO:0009266	response to temperature stimulus	0.000863101	0.0585092	1.57087
GO:0016051	carbohydrate biosynthetic process	0.00102813	0.0650642	1.66592
GO:0043255	regulation of carbohydrate biosynthetic process	0.00106083	0.0650642	1.86778
GO:0010286	heat acclimation	0.0012604	0.0737909	1.89818
GO:0009832	plant-type cell wall biogenesis	0.00186757	0.100289	1.75285
GO:0009860	pollen tube growth	0.00186874	0.100289	1.7606
GO:0009737	response to abscisic acid	0.0019945	0.102757	1.50051
GO:0048588	developmental cell growth	0.00250635	0.116199	1.70688
GO:0097305	response to alcohol	0.00257972	0.116199	1.51156
GO:0005976	polysaccharide metabolic process	0.00258596	0.116199	1.57273
GO:0072329	monocarboxylic acid catabolic process	0.00263883	0.116199	1.79327
GO:0048868	pollen tube development	0.00270649	0.116199	1.70688

Table S5. Top upstream transposable element-associated candidate driver genes showing enhanced drought-responsive expression. Genes were ranked according to the interaction effect between drought and well-watered treatments, calculated as the difference in expression change between T3 and T1 under drought relative to well-watered conditions (Interaction effect = log_2FC_Drought – log_2FC_Wellwatered). Only genes associated with upstream transposable element-linked CHH differentially methylated positions (DMPs) and showing positive interaction effects were retained. The table includes genomic feature class, gene identifier, Arabidopsis gene used in TAIR orthologues, Arabidopsis symbol, mean log_2 fold changes under drought and well-watered conditions, interaction effect size, Gene Ontology annotation, the number of associated CHH DMPs (DMP.n), and whether there is strong or medium evidence for a putative role in drought-response. Genes are ordered by decreasing interaction effect size within feature class.

gene	at_gene	SYMBOL	logFC_Drought	logFC_Wellwatered	Interaction_effect	description	DMP.n	Strong drought evidence	Moderate drought evidence	GSEA leading edge terms
LOC115976457	AT2G32300	UCC1	1.03706	-6.03055	7.06761	Uclacyanin 1 {ECO:0000303|PubMed:9761472}	3			
LOC115968716	AT2G16700	ADF5	5.29819	-1.61476	6.91295	Actin-depolymerizing factor 5	2	Liu et al., 2024		
LOC115981892	AT1G68725	AGP19	2.80624	-4.0505	6.85673	Lysine-rich arabinogalactan protein 19	12			cell wall polysaccharide biosynthetic process | pectin biosynthetic process | cell wall macromolecule metabolic process | polysaccharide biosynthetic process | cell wall polysaccharide metabolic process | cell wall pectin biosynthetic process | carbohydrate biosynthetic process
LOC115958076	AT2G04570	GGL14	0.884931	-4.79	5.67494	GDSL esterase/lipase At2g04570	6	Xiao et al., 2021		
LOC115978043	NA	NA	1.9377	-3.73411	5.67182	Protein TRACHEARY ELEMENT DIFFERENTIATION-RELATED 7A	6			

gene	at_gene	SYMBOL	logFC_Drought	logFC_Wellwatered	Interaction_effect	description	DMP.n	Strong drought evidence	Moderate drought evidence	GSEA leading edge terms
						{ECO:0000303lPubMed:19383897}				
LOC115957395	AT1G01120	KCS1	0.879577	-4.78581	5.66538	3-ketoacyl-CoA synthase 1 {ECO:0000303lPubMed:10074711}	1	Todd et al., 1999		carboxylic acid metabolic process l organic acid metabolic process l oxoacid metabolic process l monocarboxylic acid metabolic process l wax biosynthetic process l wax metabolic process l response to temperature stimulus
LOC115958173	NA	NA	4.32006	-1.04985	5.36991	NAC domain-containing protein JA2L {ECO:0000305}	12	Du et al., 2014		
LOC115963799	AT2G43330	ATINT1	3.73308	-1.30786	5.04094	Inositol transporter 1	9		Schneider et al., 2008	
LOC115951359	AT5G01360	TBL3	0.50388	-4.34233	4.84621	Protein trichome birefringence-like 3	11			cell wall polysaccharide biosynthetic process l pectin biosynthetic process l cell wall macromolecule metabolic process l polysaccharide biosynthetic process l cell wall polysaccharide metabolic process l xylan metabolic

gene	at_gene	SYMBOL	logFC_Drought	logFC_Wellwatered	Interaction_effect	description	DMP.n	Strong drought evidence	Moderate drought evidence	GSEA leading edge terms
										process l carbohydrate biosynthetic process
LOC115964092	AT1G20440	AtCOR47	4.01804	-0.645041	4.66308	Dehydrin COR47	21	Nylander et al., 2001		response to temperature stimulus l heat acclimation
LOC115995164	AT5G67130	NA	2.68965	-1.9687	4.65835	PI-PLC X domain-containing protein At5g67130	3	Hirayama et al.,1995		
LOC115959738	AT5G32470	PALE1	4.54843	-0.0867983	4.63523	Bifunctional TH2 protein, mitochondrial {ECO:0000303lPubMed:27677881}	8			
LOC115986978	AT4G21510	AtFBS2	4.7271	0.223598	4.5035	F-box protein SKIP27	9			
LOC115987405	NA	NA	2.37921	-2.12239	4.5016	.	7			
LOC115954652	AT3G15050	IQD10	2.69015	-1.75682	4.44697	Protein IQ-DOMAIN 10 {ECO:0000303lPubMed:16368012}	6			
LOC115992069	NA	NA	-0.0402595	-4.22582	4.18556	Pectinesterase inhibitor	2	Hyun An et al., 2008		
LOC115958477	NA	NA	2.50909	-1.62238	4.13147	.	12			
LOC115974406	AT4G34610	BLH6	2.03142	-2.06212	4.09355	BEL1-like homeodomain protein 6	24	Zhang et al., 2024		
LOC115983349	AT2G28315	UXT1	1.60961	-2.4601	4.06971	UDP-xylose transporter 1 {ECO:0000312lEMBL:AKA88212.1}	17			pectin biosynthetic process l polysaccharide biosynthetic process l carbohydrate biosynthetic process l regulation of carbohydrate biosynthetic process
LOC115971729	AT1G02660	PLIP2	2.94931	-1.12019	4.0695	Phospholipase A1 PLIP2, chloroplastic {ECO:0000305}	3	Wang et al., 2018		
LOC115954589	AT4G01470	ATTIP1.3	2.61676	-1.2674	3.88416	Aquaporin TIP1-3	20		Afsal et al., 2014	

gene	at_gene	SYMBOL	logFC_Drought	logFC_Wellwatered	Interaction_effect	description	DMP.n	Strong drought evidence	Moderate drought evidence	GSEA leading edge terms
LOC115950265	NA	NA	2.80381	-1.04246	3.84626	.	16			
LOC115974544	NA	NA	2.11187	-1.4918	3.60367	Probable nucleoredoxin 2	5	Zhang et al., 2021		
LOC115960690	AT5G52640	ATHS83	2.40692	-1.16739	3.5743	Heat shock protein 90-1 {ECO:0000305}	2		Clement et al., 2011	regulation of protein stability | response to temperature stimulus
LOC115975961	AT3G12610	DRT100	3.71206	0.157646	3.55441	DNA damage-repair/toleration protein DRT100	8			
LOC115952209	AT3G04070	ANAC047	2.66645	-0.877645	3.5441	NAC transcription factor 47 {ECO:0000305}	1		Singh et al., 2021	carboxylic acid metabolic process | organic acid metabolic process | oxoacid metabolic process
LOC115976498	NA	NA	2.19713	-1.27488	3.47201	.	21			
LOC115968192	NA	NA	3.43379	-0.0146786	3.44847	.	2			
LOC115953117	NA	NA	2.3291	-1.02111	3.35021	Delta-1-pyrroline-5-carboxylate synthase	5	Zhang et al., 1997		
LOC115965444	NA	NA	3.89035	0.547948	3.3424	Myrcene synthase, chloroplastic	20			

Table S6. Methylation machinery genes response to drought.

gene	mean_logFC_D	mean_logFC_WW	estimate	p_raw	tair	symbol	role	description
LOC115978625	-0.278145	0.00382793	-0.281973	0.0197462	AT2G35160	SUVH5	H3K9me2	Histone-lysine N-methyltransferase, H3 lysine-9 specific SUVH5
LOC115989792	-0.164611	-0.632728	0.468116	0.0268204	AT4G20910	HEN1	small-RNA methylation	Small RNA 2'-O-methyltransferase
LOC115988128	0.209642	-0.0681564	0.277799	0.114981	AT1G57820	VIM1	UHRF1-like, CG maintenance	E3 ubiquitin-protein ligase ORTHRUS 2
LOC115959155	0.362158	0.805881	-0.443723	0.157769	AT2G35160	SUVH5	H3K9me2	Histone-lysine N-methyltransferase, H3 lysine-9 specific SUVH5
LOC115963274	0.766247	-0.484887	1.25113	0.214657	AT5G49160	MET1	CG maintenance	DNA (cytosine-5)-methyltransferase 1
LOC115971226	-0.0388017	-0.834229	0.795427	0.217351	AT2G22740	SUVH6	H3K9me2	Histone-lysine N-methyltransferase, H3 lysine-9 specific SUVH6
LOC115978847	-0.577464	-0.138655	-0.43881	0.222569	AT5G14620	DRM2	CHH de novo (RdDM primary)	DNA (cytosine-5)-methyltransferase DRM2 {ECO:0000305}
LOC115952978	0.0928271	-0.66165	0.754477	0.225	DML_family	DML_pool	active demethylation (paralog pool)	Transcriptional activator DEMETER
LOC115994898	0.508428	-0.233867	0.742295	0.23072	AT3G17310	DRM3	RdDM regulator	Probable inactive DNA (cytosine-5)-methyltransferase DRM3 {ECO:0000305}
LOC115963743	-0.130347	-0.904924	0.774577	0.257126	AT1G57820	VIM1	UHRF1-like, CG maintenance	E3 ubiquitin-protein ligase ORTHRUS 2
LOC115982515	-0.473959	-0.116233	-0.357726	0.261682	AT2G27040	AGO4	RdDM small-RNA effector	Protein argonaute 4 {ECO:0000303lPubMed:12522258, ECO:0000303lPubMed:28229965}
LOC115982033	0.650341	0.261008	0.389333	0.290458	AT1G57820	VIM1	UHRF1-like, CG maintenance	E3 ubiquitin-protein ligase ORTHRUS 2
LOC115981714	1.05262	-0.351357	1.40398	0.295279	DML_family	DML_pool	active demethylation (paralog pool)	Transcriptional activator DEMETER
LOC115989244	-0.140859	0.230747	-0.371606	0.32775	AT4G11130	RDR2	RdDM RNA-dep RNA polymerase	RNA-dependent RNA polymerase 2 {ECO:0000303lPubMed:15024409, ECO:0000303lPubMed:15692015}
LOC115971602	-0.840355	-0.402487	-0.437868	0.357381	AT4G19020	CMT2	CHH heterochromatin + CHG	DNA (cytosine-5)-methyltransferase CMT2
LOC115986551	0.588802	0.0358144	0.552988	0.422376	DML_family	DML_pool	active demethylation (paralog pool)	Transcriptional activator DEMETER
LOC115958831	-0.110421	0	-0.110421	0.42265	AT2G35160	SUVH5	H3K9me2	Histone-lysine N-methyltransferase, H3 lysine-9 specific SUVH5

gene	mean_logFC_D	mean_logFC_WW	estimate	p_raw	tair	symbol	role	description
LOC115981080	-0.105493	0.0793236	-0.184817	0.445811	AT2G35160	SUVH5	H3K9me2	Histone-lysine N-methyltransferase, H3 lysine-9 specific SUVH5
LOC115994684	-0.183148	0.305001	-0.488149	0.459345	AT5G66750	DDM1	chromatin remodeler	ATP-dependent DNA helicase DDM1
LOC115969816	-0.257763	-0.722491	0.464729	0.561611	AT1G63020	NRPD1	Pol IV largest subunit	DNA-directed RNA polymerase IV subunit 1
LOC115986796	-0.431787	-0.135102	-0.296685	0.579795	AT2G40030	NRPE1	Pol V largest subunit	DNA-directed RNA polymerase V subunit 1
LOC115969414	-0.153689	0.00048	-0.154169	0.612399	DML_family	DML_pool	active demethylation (paralog pool)	Protein REPRESSOR OF SILENCING 3 {ECO:0000303lPubMed:18815596}
LOC115950261	0.028139	0.189406	-0.161267	0.615489	AT2G22740	SUVH6	H3K9me2	Histone-lysine N-methyltransferase, H3 lysine-9 specific SUVH6
LOC115963412	-0.483467	-0.147159	-0.336309	0.704701	AT4G11130	RDR2	RdDM RNA-dep RNA polymerase	RNA-dependent RNA polymerase 2 {ECO:0000303lPubMed:15024409, ECO:0000303lPubMed:15692015}
LOC115982048	0.088507	-0.0937537	0.182261	0.74516	AT5G13960	SUVH4	H3K9me2 (KYP)	Histone-lysine N-methyltransferase, H3 lysine-9 specific SUVH4
LOC115978052	0.130018	0.351804	-0.221786	0.777778	AT1G69770	CMT3	CHG maintenance	DNA (cytosine-5)-methyltransferase CMT3 {ECO:0000305}
LOC115976353	-0.297839	-0.226442	-0.0713977	0.816278	AT5G13960	SUVH4	H3K9me2 (KYP)	Histone-lysine N-methyltransferase, H3 lysine-9 specific SUVH4
LOC115953674	0.560787	0.420231	0.140556	0.833996	AT3G43920	DCL3	RdDM Dicer	Endoribonuclease Dicer homolog 3
LOC115976632	0.0245991	-0.0687699	0.0933691	0.853586	AT5G14620	DRM2	CHH de novo (RdDM primary)	DNA (cytosine-5)-methyltransferase DRM2
LOC115960480	-0.0599801	-0.163497	0.103517	0.890743	AT2G35160	SUVH5	H3K9me2	Histone-lysine N-methyltransferase, H3 lysine-9 specific SUVH5
LOC115953675	0.555247	0.489741	0.0655058	0.895172	AT3G43920	DCL3	RdDM Dicer	Endoribonuclease Dicer homolog 3
LOC115953111	0.411908	0.443321	-0.0314137	0.953022	AT3G43920	DCL3	RdDM Dicer	Endoribonuclease Dicer homolog 3
LOC115989355	0.141078	0.134023	0.00705482	0.98823	AT1G63020	NRPD1	Pol IV largest subunit	DNA-directed RNA polymerase IV subunit 1
